# The structure of an actin nucleus stabilized by villin

**DOI:** 10.1126/sciadv.adw6915

**Published:** 2025-12-03

**Authors:** Robert C. Robinson, Thitipat Chongrungreang, Khongpon Ponlachantra, Bundit Boonyarit, Geoffrey F. Dilly, Yang I. Li, Peter R. Girguis, Richard R. Copley, Adam Claridge-Chang

**Affiliations:** ^1^ School of Biomolecular Science and Engineering (BSE), Vidyasirimedhi Institute of Science and Technology (VISTEC), Rayong 21210 Thailand.; ^2^ Research Institute for Interdisciplinary Science (RIIS), Okayama University, Okayama 700-8530, Japan.; ^3^ School of Information Science and Technology (IST), Vidyasirimedhi Institute of Science and Technology (VISTEC), Rayong 21210, Thailand.; ^4^ Department of Biology, California State University Channel Islands, 1 University Drive, Camarillo, CA 93012, USA.; ^5^ Section of Genetic Medicine, Department of Medicine, University of Chicago, Chicago, IL 60637, USA.; ^6^ Department of Organismic and Evolutionary Biology, Harvard University, Cambridge, MA 02138, USA.; ^7^ CNRS, Laboratoire de Biologie du Développement de Villefranche-sur-mer (LBDV), Sorbonne Université, Villefranche-sur-Mer, France.; ^8^ Program in Neuroscience and Behavioral Disorders, Duke-NUS Medical School, Singapore 138673, Singapore.

## Abstract

Villin is an actin filament nucleating, severing, capping and bundling protein; however, the structural basis for villin’s functions and the characteristics of the actin polymerization nucleus remain poorly understood. Here, we present the structure of vent-worm villin bound to a trimeric actin nucleus. Villin wraps around and caps the barbed end of the actin trimer. Its headpiece domain interacts at the junction of two laterally associated actin protomers, leaving the pointed-end subunits open for elongation. Within the actin trimer, the two longitudinally associated subunits adopt barbed and pointed-end subunit conformations, while the lateral protomer exhibits a monomeric conformation. This provides the first view of an actin-filament nucleus, revealing that the transition into the filamentous form is stimulated and stabilized by the interactions with the pointed-end subunits. Our results also illuminate mechanisms of actin-filament dynamics and villin capping and severing, suggesting that F-to-G actin conformational transitions facilitate the later process.

## Teaser

Villin’s structure with an actin trimer uncovers the earliest steps of actin filament formation and the basis of severing.

## INTRODUCTION

The actin cytoskeleton is composed of a single-filament architecture (*1*) that assembles into various structures essential for cell shape, dynamics, and organization in all eukaryotes (*2*). In isolation, actin filament assembly begins with the longitudinal dimerization of monomers, followed by the lateral association of a third subunit to form a three-subunit nucleation core (*3*–*5*). The conformations of the actin subunits within this nucleation core remain unknown. Subsequent filament elongation proceeds more rapidly at the barbed end than at the pointed end. Conversely, when actin monomer concentrations favor depolymerization, filament dissociation occurs more quickly at the barbed end compared to the pointed end (*6*). Within cells, actin-interacting proteins enhance and regulate these innate actin properties—for example, formins accelerate nucleation; profilins further bias barbed-end elongation; and cyclase-associated protein depolymerizes filaments (*2*, *7*–*9*). Beyond the intrinsic properties of actin, other regulating proteins bring additional properties, such as filament severing (cofilin), capping (caping protein), uncapping (twinfilin), and bundling (fascin), leading to the wide variety of actin dynamics and architectures observed in cells (*10*).

Villin is a member of the calcium-regulated gelsolin family of actin filament severing and capping proteins, a class of proteins found in Asgard archaea and nearly all eukaryotes (*11*–*13*). These proteins contain gelsolin-homology (G) domains consisting of ~100 to 120 residues arranged in a β sheet sandwiched between two α helices (*14*), with three to six G domains present in eukaryotic gelsolin family proteins and one or two G domains in Asgard archaean proteins (*12*, *13*). Eukaryotic G domains are often associated with a ~65-residue C-terminal α-helical headpiece (HP) domain, indicating that this architecture was likely present in the last eukaryotic common ancestor (*12*). Humans express eight G domain–containing proteins, three of which exhibit a typical villin-like structure featuring six G domains (referred to here as V domains) followed by an HP domain (*14*). In vitro, villin can nucleate actin polymerization and bundle filaments (*15*, *16*). However, in the cell, where profilin prevents pointed-end elongation (*17*), villin’s roles involve severing and capping filaments upon calcium stimulation (*18*). While gelsolin has been well characterized structurally and functionally, with its calcium-bound conformation exposing actin-binding sites to sever and cap filaments (*19*–*23*), the roles of villin and, particularly, of the HP domain remain unclear. Furthermore, although the structures of G-actin, F-actin, and actin’s barbed and pointed ends are known (*24*, *25*), the structure of the actin nucleus is elusive. To address these issues, we used x-ray crystallography to understand how villin binds to an actin trimer.

## RESULTS

### Structure of villin bound to an actin trimer

Our previous attempts to crystallize mammalian villin/actin complexes were unsuccessful, prompting a shift to exploring villins from thermotolerant species. We focused on a villin sequence derived from the deep-sea worm *Paralvinella sulfincola* (*P. sul*; fig. S1), a species inhabiting thermal vents of the Pacific Ocean, with the hypothesis that its inherent thermal stability would enhance crystallization efforts. We successfully crystallized a complex of *P. sul* villin bound to three subunits of adenosine 5′-diphosphate (ADP)-Mg^2+^-actin (VA3).

**Fig. 1.**
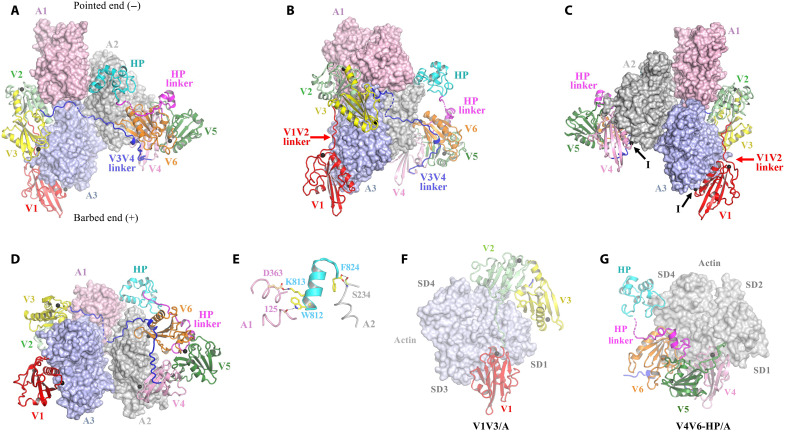
Villin-stabilized actin nucleus. (**A**) Front, (**B**) side, (**C**) back, and (**D**) barbed-end views of the tetrameric complex. Villin is shown in cartoon representation and consists of seven domains: V1 (red), V2 (light green), V3 (yellow), V4 (pink), V5 (dark green), V6 (orange), and HP (cyan). Extended linker regions are present between V1V2 (red arrow), V3V4 (blue), and V6-HP (magenta). The actin subunits are shown in surface representation and are labeled A1 (pink), A2 (gray), and A3 (slate blue), numbered sequentially from the pointed to barbed ends. The eight calcium ions are shown as black spheres; the two type I calcium ions sandwiched between villin and actin are indicated by “I” in (C). The arrangement of V domains is similar to that of G domains in the cryo-EM structure of the gelsolin-capped filament (movie S2) (*22*). (**E**) Close-up of the C-terminal helix of the villin HP domain, which binds at the intersection of two laterally related actin subunits, A1 and A2. Residues involved in this interaction are depicted as sticks. (**F**) V1V3:actin structure. (**G**) V4V6-HP:actin structure.

The resulting structure was refined against 2.7-Å-resolution data and is characterized by *R* factors of 19.6 (work) and 22.6 (free) (table S1). The asymmetric unit contains two tetrameric complexes, each of which reveals a stable three–actin subunit nucleus, with villin playing a critical role in its stabilization (Fig. 1, A to D, and movie S1). Specifically, V1 binds to the barbed-end actin (A3), and the V1V2 linker extends up this subunit; this facilitates V2V3 domain interactions at the intersection with the longitudinally associated actin subunit (A1), which represents the pointed end of the actin nucleus. The V3V4 linker encircles the actin nucleus, enabling V4 to interact with the laterally associated subunit (A2). Notably, A2 corresponds to the second actin subunit at both the pointed and barbed ends of the nucleus, with V4 engaging A2 similarly to how V1 interacts with A3. The V3V4 linker also binds to V6, stabilizing V4V6 in an active conformation and allowing both V4 and V6 to contact A2. The HP linker facilitates binding of the HP domain at the junction between A1 and A2 (Fig. 1E). Within this villin-stabilized actin nucleus, the V2V3 domains reinforce the interactions of actins A1 and A3, while the V3V4 linker and HP domain stabilize the interactions of A2 with both A1 and A3. Villin associates with eight calcium ions: one type II ion in each of the six V domains and one type I ion apiece in the V1/A3 and V4/A2 interfaces (Fig. 1C) (*22*). The relative positions of the villin domains (V1 to V6) closely resemble the gelsolin domains (G1 to G6) from the cryo–electron microscopy (cryo-EM) structure of the gelsolin-capped filament (fig. S2 and movie S2) (*22*).

### Actin conformations

**Fig. 2.**
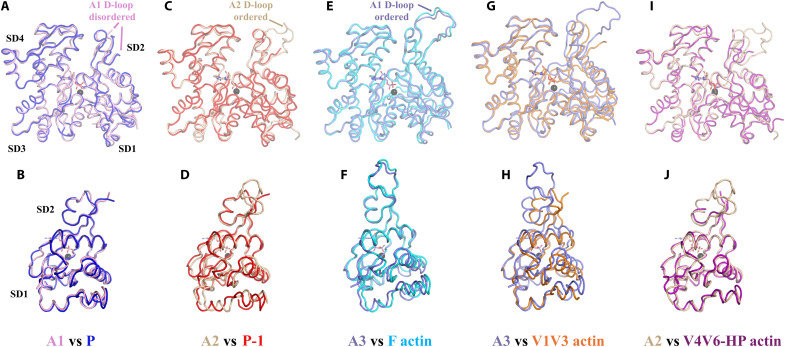
Actin conformational states in the VA3 complex. (**A** and **B**) Front and side views of the superimposition of actin subunit A1 (pink) with the cryo-EM structure of the terminal actin subunit at the pointed end [P, blue, PDB ID 8F8S, root mean square deviation (RMSD) of 0.7 Å for 359 residues] (*21*). The four subdomains (SD1 to SD4) are labeled. (**C** and **D**) Comparison of actin subunit A2 (tan) with the proximal terminal actin subunit at the pointed end (P-1, red, PDB ID 8F8S, RMSD of 0.9 Å for 350 residues) (*21*). (**E** and **F**) Comparison of actin subunit A3 (slate blue) with the cryo-EM structure of the barbed-end F-actin subunit (cyan, PDB ID 8F8R, RMSD of 0.8 Å for 370 residues) (*25*). (**G** and **H**) Comparison of actin A3 (slate blue) with the G-actin structure from the V1V3/actin complex (orange, RMSD of 1.4 Å for 351 residues). (**I** and **J**) Comparison of actin subunit A2 (tan) with the G-actin structure from the V4V6-HP/actin complex (magenta, RMSD of 0.9 Å for 346 residues). The VA3-bound ADP and cations are shown as sticks and spheres (black), respectively. All superimpositions were generated by overlaying actin SD3 and SD4 to observe the relative changes in SD1 and SD2. SD3 and SD4 are omitted from the side views for clarity. An animated version of the actin conformational states is shown in movie S3.

Within the actin nucleus, subunit A1 adopts a conformation that exhibits close structural homology to the pointed-end subunits of F-actin (Fig. 2, A and B; fig. S3A; and Table 1). Subunit A1 is characterized by a subdomain 1 (SD1)–to–SD4 dihedral angle of −22.7° similar to those of the twisted G-actin and the pointed-end subunits of F-actin, −23.6° and ~−22.0°, respectively (Table 1). However, the W-loop is in the open conformation to accept the D-loop residue Met^44^ from subunit A3 (Y143-to-Y169 Cα distance, 12.6 Å; Y143 OH–to–G168 O distance, 12.6 Å; Table 1 and movie S3), more similar to a pointed-end subunit than G-actin (*4*).

**Table 1.** Structural relatedness of actin subunits. Top section: RMSDs with the number of matched Cα positions are given for each pairwise structural comparison. VA3 actin A3 groups with F-actin conformations. B actin, B-1 actin, and F-actin refer to the sequential actin subunits from the cryo-EM structure of the actin filament barbed end (PDB ID 8F8R) (*25*). VA3 actins A1 and A2 group with G-actin conformations. P actin and P-1 actin refer to the sequential actin subunits from the cryo-EM structure of the actin filament pointed end (PDB ID 8F8S) (*25*). V1V3 and V4V6-HP refer to the actin subunits bound to these fragments reported here. G1G3 (PDB ID 3FFK) and G4G6 (PDB ID 1H1V) refer to the corresponding actin subunits from gelsolin/actin x-ray structures (*23*, *45*). G-actin is the native actin x-ray structure (PDB ID 3HBT) (*24*). Gelsolin B actin1 refers to the barbed-end actin subunit bound to G1 from the structure of the cryo-EM structure of the gelsolin-capped filament (PDB ID 8VIZ) (*22*). “Distances and angles” section, analysis of angles and distances in the actin subunits. Center-of-mass calculations were used to generate the distances and angles between subdomains (fig. S19). The distance between Tyr^143^ and Tyr^169^ Cα atoms indicates the movement of the W-loop (*4*), and the distance between Tyr^143^ (OH) and Gly^168^ (O) indicates the opening and closing of the W-loop gate. Numbers in parentheses indicate distances from an alternate cryo-EM structure of the barbed end (PDB ID 8RU0) (*28*). ϕ SD1–SD4 indicate the dihedral angles. Δ SD1 and Δ SD2 indicate the relative offsets of the center of masses of SD1 and SD2 relative to G-actin and F-actin (fig. S19).

	G-actin–like conformations									F-actin–like conformations				
Structure	A1	A2	P actin	P-1 actin	V1V3	V4V6	G1G3	G4G6	G-actin	A3	B actin	B-1 actin	F-actin	Gelsolin B actin1
A1	0.0	0.9	0.7	0.6	1.0	0.7	1.1	1.0	1.1	2.0	2.2	2.3	2.3	1.7
	361	350	359	359	349	348	354	360	359	361	359	360	361	361
A2	0.9	0.0	1.0	0.9	0.8	0.9	1.9	0.8	1.0	2.0	2.0	2.2	2.1	1.8
	350	363	349	350	350	346	359	357	349	358	356	358	357	358
V1V3	1.0	0.8	1.1	1.3	0.0	0.9	0.7	0.7	1.2	1.4	1.5	1.6	1.6	1.1
	349	350	349	351	351	344	351	351	350	351	351	351	351	351
V4V6	0.7	0.9	0.9	0.8	0.9	0.0	1.0	0.8	0.9	1.7	1.8	1.9	1.9	1.5
	348	346	347	347	344	351	349	347	347	348	347	348	348	348
A3	2.0	2.0	2.1	2.1	1.4	1.7	3.1	1.7	2.3	0.0	0.8	0.9	0.9	0.8
	361	358	359	360	351	348	364	368	359	372	370	371	372	372
Distances and angles														
ϕ SD1–SD4, °	−22.7	−17.7	−21.8	−22.1	−16.9	−19.9	−14.8	−16.3	−23.6	−8.1	−6.9	−6.4	−6.2	−10.9
Δ SD2 G-actin, Å	1.3	1.3	0.9	0.9	3.3	1.5	3.5	1.9	–	7.5	7.1	7.1	7.5	6.8
Δ SD2 F-actin, Å	6.8	6.2	6.8	6.8	4.2	6.4	4.1	5.9	7.5	0.5	0.7	0.6	–	1.1
Δ SD1, °	1.6	3.2	1.3	1.2	2.4	2.6	1.2	2.6	–	4.9	4.3	4.5	5.5	3.6
G-actin, Å	0.9	1.6	0.7	0.8	1.3	1.3	0.6	2.0	–	2.5	2.1	2.2	2.7	2.0
Δ SD1, °	3.9	4.4	4.4	4.2	3.5	4.0	4.2	4.1	5.5	1.7	1.5	1.0	–	3.9
F-actin, Å	1.9	2.2	2.2	2.1	1.5	2.0	2.1	3.1	2.7	0.9	0.8	0.8	–	2.0
Y143 CA and Y169 CA, Å	12.6	11.2	12.9	13.3	11.6	11.0	10.5	10.9	11.2	11.1	11.3 (11.4)	11.8 (12.1)	13.0	12.0
Y143 OH and G168 O, Å	12.6	3.5	12.1	11.3	3.2	3.5	2.9	3.7	11.6	3.6	3.8 (5.8)	4.1 (6.2)	7.3	3.8

Subunit A2 adopts a similar yet slightly flattened structure by 5°, with an SD1-to-SD4 dihedral angle of −17.7° (Fig. 2, C and D; fig. S3B; and Table 1). However, the W-loop is in the closed conformation (Y143-to-Y169 Cα distance, 11.2 Å; Y143 OH–to–G168 O distance, 3.5 Å; Table 1 and movie S3) (*4*), like most G-actin structures. The D-loop of subunit A2 is ordered and compact, forming interactions between the two copies of the VA3 complex in the crystal asymmetric unit (fig. S4A). This D-loop conformation is not suitable to form longitudinal or lateral contacts within a filament (fig. S4, B and C), and we propose that it is induced by the crystal contacts.

**Fig. 3.**
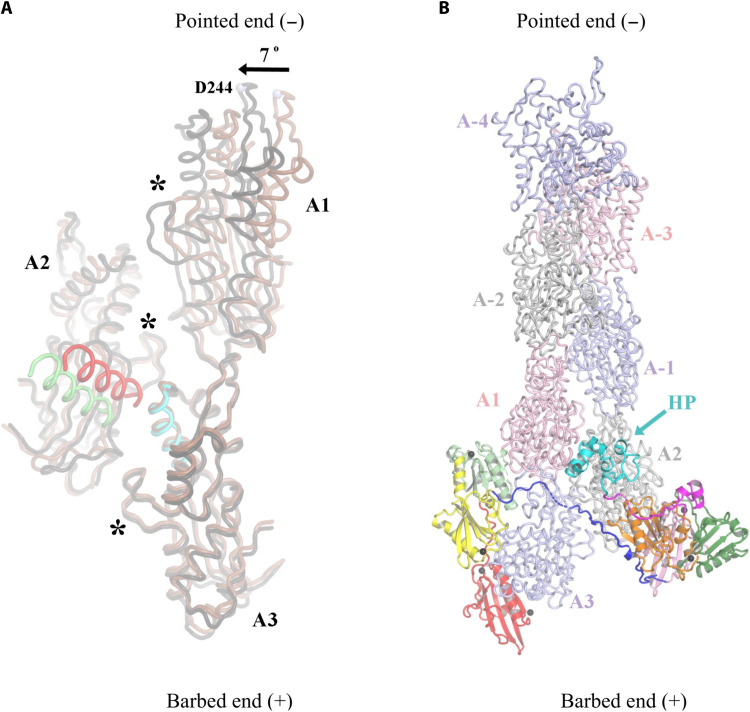
Comparison of the villin-stabilized actin nucleus with F-actin. (**A**) Comparison of the three actin subunits from VA3 (brown) with F-actin (black, PDB ID 8A2T) (*1*). Only SD3 and SD4 are shown for clarity. Asterisks indicate the H-loops, which form interstrand interactions. The second interstrand interaction in F-actin via the helix zone (red and cyan helices) is not present in VA3 (green helix), given that A2 adopts a conformation similar to G-actin (Table 1). A1 is tilted in the VA3 structure and would need to tilt by 7°, measured at Asp^244^, in the direction of the arrow to adopt the position of the corresponding F-actin subunit. (**B**) Villin is placed onto the barbed end of F-actin by superimposing actin subunit A3 from the VA3 structure onto the terminal actin subunit of the barbed end of a filament (PDB ID 8F8R) (*25*). The VA3 actin subunits are omitted. The villin HP domain exclusively contacts actin subunits A1 and A2.

By contrast, actin subunit A3 shares many features of the flattened F-actin and barbed-end subunits (Fig. 2, E and F; Table 1; and movie S3). However, the W-loop is in the closed conformation (Y143-to-Y169 Cα distance, 11.1 Å; Y143 OH–to–G168 O distance, 3.6 Å; Table 1) (*4*). This subunit is characterized by an SD1-to-SD4 dihedral angle of −8.1°, close to fully flattened F-actin at −6.2°. The D-loop of subunit A3 is ordered and extended, allowing it to interact with subunit A1 in a similar manner to two longitudinal subunits in a filament (fig. S5, A and B), and the A1/A3 buried area (~1198 Å^2^) is similar to the buried area of longitudinally related subunits in the cryo-EM structures of actin filaments [1064 to 1214 Å^2^, calculated from Protein Data Bank (PDB) IDs 8F8R and 8A2T] (*1*, *25*). However, we observed subunit A1 to be tilted away from the F-actin orientation by 7°, corresponding to a displacement of 8.3 Å at the SD4 residue Asp^244^ (Fig. 3A).

The H-loop of subunit A2 forms lateral interactions with SD2 and SD3 from subunits A3 and A1 with buried areas of 196 and 80 Å^2^, respectively, similar to the H-loop lateral interactions observed in a filament (Fig. 3A and fig. S6). However, the interactions in the lateral helix zone contact between SD4, which are found in the filament (fig. S6), are not present in the VA3 complex (Fig. 3A). These would require a flattened conformation of A2 to support simultaneous interactions of the H-loop and lateral helix zone across the complex (*26*).

### HP interactions

The C-terminal helix of the HP domain fits snugly between the actin SD1 and SD4 from A1 and A2, respectively (Fig. 1E and fig. S7, A and B). Here, the C-terminal carboxyl group (Phe^824^) forms hydrogen bonds with the side chain and the backbone nitrogen of Ser^234^ from A2. In addition, residues Trp^812^ and Lys^813^ of the HP C-terminal helix establish hydrogen bonds with the carbonyl oxygen of A1’s residue 125 and the side chain of Asp^363^, respectively. These interactions indicate that the HP helix functions as both a stabilizer and a sensor of the lateral association between A1 and A2.

The structure of the dematin HP has been elucidated to be bound to two actin subunits within the larger spectrin-actin junctional complex (*27*). The dematin HP uses equivalent residues Trp^393^, Lys^394^, and Phe^405^ to villin residues Trp^812^, Lys^813^, and Phe824, respectively, to interact between laterally related actin subunits, indicating that these residues are critically important to bind at the junction of actin subunits, as they are conserved in different molecular architectures (fig. S7C).

### Pointed-end elongation from VA3

**Fig. 4.**
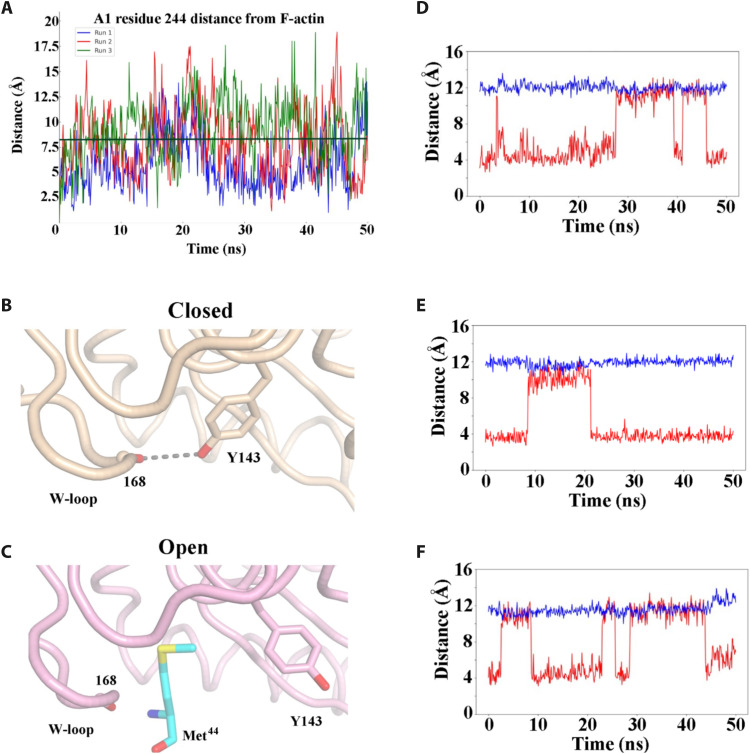
MD simulations and the W-loop gate. (**A**) Analysis of three 50-ns MD simulations of the actin trimer measuring the distance between Asp^244^ in A1 to its F-actin position after A3 is superimposed on the filament. The black horizontal line indicates the distance in (A). (**B**) Depiction of the W-loop gate in the closed (A2) and (**C**) open conformations (A1). Met^44^ (cyan) lies in the A3 D-loop. (**D** to **F**) Analysis of the Tyr^143^ distances in examples of the 50-ns MD simulations of the actin trimer for A2 and A3 and an actin monomer, respectively. Red trace, W-loop gate distance of Tyr^143^ OH to Gly^168^ O. At ~4 Å, the gate is shut, and at 8 to 12 Å, the gate is open. Blue trace, width of the Met^44^ binding pocket, Tyr^143^ Cα–to–Tyr^169^ Cα distance.

Next, we explored the mechanism of pointed-end elongation, from the villin-stabilized actin nucleus, by superimposing the VA3 complex onto the structure of the actin filament (Fig. 3, A and B, and movie S1) (*25*). The villin/actin interactions remained confined to those present in VA3 (Fig. 1A). The HP domain interacts exclusively with actin subunits A1 and A2 (Fig. 3B), while V1 and V4 cap subunits A3 and A2, respectively. Consistent with the VA3 actin nucleus supporting pointed-end elongation (*17*), the positions of the VA3 actin subunits closely resemble those in the filament (Fig. 3, A and B, and movie S1). To assess whether the 7° tilt of A1 from the canonical F-actin orientation would destabilize the nucleus in the absence of villin (Fig. 3A), we performed 50-ns molecular dynamics (MD) simulations on the actin trimer alone, with ADP replaced by adenosine 5′-triphosphate (ATP). In these short simulations, the trimer remained intact, and the residue Asp^244^ of subunit A3 fluctuated within ~1.3 to 17 Å relative to its canonical filament position (Figs. 3A and 4A and movie S4). This flexibility suggests that the villin-free trimer samples conformations compatible with pointed-end elongation. Thus, villin interactions appear to stabilize the positions of the subunits in a configuration similar to a filament, and we propose that conformational dynamics enable closer lateral subunit association to support pointed-end elongation.

### Actin trimer stability

A longer MD simulation of 600 ns revealed that A2 separated from A1/A3 at ~400 ns, in line with the instability of the actin trimer and the small contact area between A1/A3 and A2 (<300 Å^2^) (movie S4) (*4*, *26*). The A1/A3 interaction remained intact via the relatively stable A3 SD4 and D-loop interactions with A1 SD3 compared to the more minor interaction with the A2 H-loop. Thus, short MD simulations reveal fluctuations in the conformations in the subunit positions, which would support filament elongation, while the longer simulation indicates that the dissociation of the trimer proceeds first through the loss of the lateral interactions to leave a longitudinal dimer, which we suggest requires a longer MD simulation to observe to dissociate.

### Fluctuations of the W-loop gate important for elongation at both ends

To assess whether the actin trimer provides a suitable model for barbed-end elongation, we analyzed W-loop distances between the Cα atoms of Tyr^143^ and Tyr^169^, as well as the H-bonding distance between the Tyr^143^ side-chain hydroxyl (OH) and Gly^168^ oxygen (O) atoms (Table 1). Tyr^143^ serves as a gatekeeper (W-loop gate) for the hydrophobic pocket that accommodates the D-loop residue Met^44^ in F-actin (Fig. 4, B and C). In the closed state, a hydrogen bond between Tyr^143^ OH and Gly^168^ O shuts the W-loop gate and access to the Met^44^ binding site (Fig. 4B), as observed in most G-actin x-ray structures (Table 1 and movie S3). In the W-loop gate open state, Try^143^ flips away to reveal the Met^44^ binding site (Fig. 4C). In the x-ray structure of native G-actin crystallized from a solution containing HSP27, Tyr^143^ adopts an open conformation, providing an experimentally determined precedent for gate opening in an actin monomer (Table 1 and movie S3) (*24*). In two of the three 50-ns MD simulations, Tyr^143^ in subunits A2 and A3 oscillated between the Gly^168^-bound (closed) state and an open flipped-away state (Fig. 4, D and E, and fig. S8, A to C), indicating that the gate to the Met^44^ pocket undergoes dynamic opening and closing in the barbed-end subunits of the trimer. A 50-ns MD simulation of G-actin also showed dynamic gating (Fig. 4F and fig. S8D). The W-loop gate was open in ~40% of conformations for both G-actin and A2 but only ~10% for A3 (fig. S8). However, the distance between the Cα atoms of Tyr^143^ and Tyr^169^ remained relatively constant in these simulations (Fig. 4, D to F, and fig. S8), indicating that the W-loop does not shift in concert with the W-loop gate opening. We propose that the displacement of Tyr^143^ alone is sufficient to permit Met^44^ binding and that W-loop rearrangement occurs via an induced-fit mechanism rather than being a prerequisite for barbed-end subunit addition from the nucleus or during pointed-end addition.

In the highest-resolution cryo-EM barbed-end filament structure (PDB ID 8RU0; at 3.08 Å), the W-loop gate is open and the Met^44^ binding pocket expanded (Table 1) (*28*). This likely results from interactions with the laterally related flattened subunits, which impose slightly different SD1 conformations on the barbed-end subunits, in contrast to A3, whose lateral partner A2 is a G-actin–like subunit (Table 1 and movie S3). An open W-loop gate in the barbed end will favor the incoming G-actin residue Met^44^ insertion, whereas in G-actin, the fluctuating W-loop intermittently obscures the Met^44^ pocket, likely slowing addition at the pointed end and nucleation.

### Structure of the villin halves bound to actin monomers

To investigate whether the actin conformations in VA3 are dictated by villin interactions or by the formation of an actin nucleus, we solved the crystal x-ray structures of the separated three N-terminal domains V1, V2, and V3 (V1V3) and the four C-terminal domains V4, V5, V6, and HP (V4V6-HP) of villin, both in complex with a single actin subunit (Fig. 1, F and G; figs. S9 to S12; and table S1). The actin bound to V1V3 adopts an intermediate conformation that is closer to the twisted A1, A2, G-actin, and pointed-end actin structures than to the flattened A3 or the barbed-end and F-actin structures (Fig. 2, G and H; fig. S3C; Table 1; and movie S3), with an SD1-to-SD4 dihedral angle of −16.9° and a closed W-loop gate. The actin associated with V4V6-HP adopts a twisted G-actin–like structure similar to A2 (Fig. 2, I and J; fig. S3D; Table 1; and movie S3), with an SD1-to-SD4 dihedral angle of −19.9° and a closed W-loop gate. Notably, both V1V3-bound and V4V6-HP–bound actins demonstrate high structural homology with the equivalent x-ray structures of the complexes from human gelsolin (fig. S13, A and B, and Table 1). Thus, the actin conformations bound to the separated halves of villin have relaxed toward the twisted G-actin conformation by 8.7° and 2.2° relative to VA3 complex subunits A3 and A2, respectively (Table 1). The structure of A3, within the VA3 complex, stands out as an outlier in adopting a flattened F-actin–like conformation (fig. S13C), suggesting that its conformation arises from interactions within the actin nucleus.

### Determinants of conformational transition in the nucleus

**Fig. 5.**
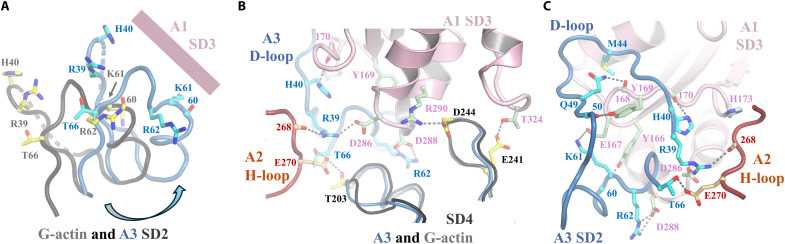
G-to-F transition inferred by the actin nucleus. (**A**) SD2 from actin A3 (slate blue) compared to the same region of G-actin (black) after superimposition of SD3 and SD4 (not shown for clarity). Key residues in the G-to-F transition are labeled. For clarity, the D-loops are omitted and replaced by dotted lines, which are largely hidden. The arrow indicates the direction of the G-to-F transition toward A1 SD3. Arg^39^ and His^40^ preceding the D-loop, Lys^61^ and Arg^62^, Thr^66^, and the carbonyl of residue 60 from the helix-turn motif following the D-loop are depicted in stick representation in actin A3 (cyan) and G-actin (yellow). (**B**) Additional residues enabling the G-to-F transition. SD4 from G-actin (black) is superimposed on actin A3 (slate blue) from the actin nucleus. A3 SD2 (slate blue) is displayed in the background. The H-loop of A2 (brown) and a section of A1 SD3 (pink) are shown. Glu^241^ and Asp^244^, from the conformationally rigid A3 SD4, interact with A1 residues Thr^234^ and Arg^290^, respectively. The position of the A2 H-loop allows residue Glu^270^ to bridge A3 residues Thr^203^ and Thr^66^ from SD4 and SD2, respectively. A3 SD2 residue Arg^39^ forms H-bonds with the carbonyl oxygen from A2 H-loop residue 268 and with A1 SD3 Asp^286^. Additional contacts are formed between A3 residues Arg^62^ and His^40^ and A1 Asp^288^ and the carbonyl oxygen of residue 170, respectively. (**C**) Further residues enabling the transition can be seen from the back view of (B). A3 SD2 Lys^61^ and the carbonyl oxygen of residue 60, from the helix-turn motif following the D-loop, bind A1 SD3 W-loop residues Glu^167^ and Tyr^166^, respectively, and Met^44^ occupying the hydrophobic pocket. The A3 D-loop locks the F-actin conformation, with Gln^49^ and the carbonyl of residue 50 contacting the A1 SD3 carbonyl of residue 168 and Tyr^169^, respectively.

To understand the determinants of the A3 F-actin conformation, we analyzed the interactions of A3 SD2 within the villin-stabilized actin nucleus by comparing it to a G-actin conformation. We superimposed G-actin onto A3 by overlaying the conformationally rigid subdomains SD3 and SD4 (Fig. 5A). A3 SD2 is shifted to the right relative to G-actin, facilitating its association with A1 SD3 (movie S5A). A closer inspection of the interactions between A3 SD2 and A1/A2 reveals that several key residues undergo substantial movement during the G-to-F transition, as explained in the following paragraph, and these residues are critical for stabilizing the F-actin structure of A3 and are candidates for inducing the G-to-F transition (*29*).

Residues Glu^241^ and Asp^244^, whether from their current positions in A3 or modeled as G-actin, are positioned to interact with Thr^324^ and Arg^290^ of A1 SD3 (Fig. 5B). This interaction places A3 Thr^203^ to engage with Glu^270^ in the A2 H-loop and for A1 SD3 His^173^ to form a van der Waals interaction with the A2 H-loop (Fig. 5, B and C, and movie S5A). We propose that the arrangement of these rigid segments—A1 SD3, A2 H-loop, and A3 SD4—prepares the binding site necessary to induce conformational changes in A3 SD2. At the base of A3 SD2, Thr^66^ sandwiches A2 H-loop Glu^270^ between itself and A3 Thr^203^, while Arg^39^ connects the carbonyl oxygen of A2 H-loop Gly^268^ to the side chain of A1 Asp^286^ (Fig. 5B and movie S5A). In addition, the carbonyl oxygen of residue 60 and the side chains of Lys^61^ and Arg^62^ form bonds with A1 W-loop residues Tyr^166^ and Glu^167^ and Asp^288^, respectively (Fig. 5, B and C, and movie S5). Once the base of A3 SD2 and the A1 W-loop are properly positioned, the A3 D-loop including Met^44^ (Fig. 4B), which requires a second actin subunit to become ordered (*30*), can access its binding site at the junction of A1 SD3 and SD1, with notable bonds between A3 His^40^, Gln^49^, and the carbonyl oxygen of residue 50 with carbonyl oxygens of residues 170 and 168 and the side chain of Tyr^169^ from the A1 W-loop, respectively (Fig. 5C and movie S5). We note that the Gly^168^ carbonyl oxygen is involved in a H-bond to secure Tyr^143^ in the closed W-loop gate (Fig. 4B); thus, interaction between the A1 Gly^168^ carbonyl oxygen and A3 Gln^49^ may also play a role in opening the gate to allow A3 Met^44^ to access its binding pocket on A1.

Thus, we propose a nucleus activation mechanism in which the G-to-F flattening transition in A3, facilitated by villin, brings together the rigid portions of A1 SD3, A2 H-loop, and A3 SD4. This arrangement provides the binding site necessary for the F-form conformation of the base of A3 SD2, positioning the A3 D-loop to interact with A1 SD3 and stabilize this conformation. We hypothesize that a similar mechanism also functions in the formation of the native actin nucleus, and this is evolutionarily conserved from Asgard archaea to eukaryotic actin (fig. S14). In summary, two longitudinally related subunits come together to induce the F-actin conformation in the subunit closest to the barbed end, which is stabilized from the side by the H-loop of the laterally related subunit, resulting in an actin nucleus that is primed for elongation (movie S5A).

## DISCUSSION

### Implications for actin nucleation and elongation

Combining structural evidence from the villin-stabilized actin nucleus with cryo-EM–determined conformations of the actin filament subunits enhances our understanding of actin polymerization dynamics in the absence of binding partners, leading to the model outlined below (*6*, *25*, *28*, *31*).

### Nucleation

**Fig. 6.**
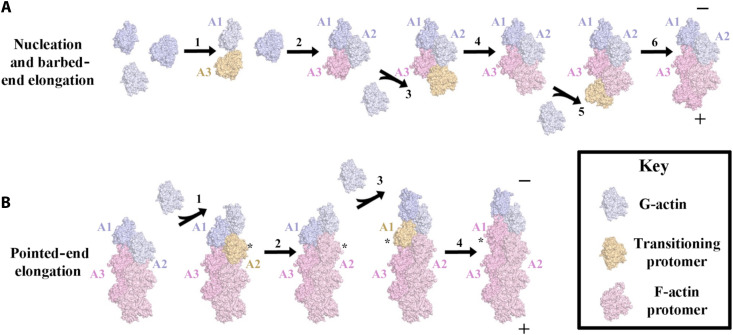
Model of actin nucleation followed by elongation from the nucleus. (**A**) Actin nucleation and subsequent barbed-end (+) elongation and (**B**) pointed-end (−) elongation. In (B), asterisks indicate the P-2 (pointed-end minus 2 subunit) actin in which the G-to-F conformational change occurs. A1 to A3 indicate the subunits from the actin nucleus. “Transitioning protomer” refers to a protomer that is undergoing the G-to-F conformational change. Movie S6 provides an animated version.

We suggest that actin filament nucleation begins with two G-actin subunits coming together in a collision complex (Fig. 6A and movie S6, step 1). Two plausible extremes of the initial contact can be envisaged: an “SD4 first” model, in which SD4 of A3 contacts SD3 of A1, or a “D-loop first” model, in which the A3 D-loop inserts into the Met^44^ binding pocket of A1. In the “SD4 first” scenario, initial A1 SD4-A3 SD3 contact is followed by A1-induced conformational changes in A3, enabling the A3 D-loop to become ordered by inserting Met^44^ into its binding site on A1 (movie S5A). In the “D-loop first” scenario, insertion of the A3 D-loop precedes a swing of A3, allowing SD4 of A3 to contact SD3 of A1 (movie S5B). MD simulations support a model in which G-actin occasionally samples conformations along the G-to-F transition pathway (*32*, *33*). Thus, while A1 may remain in a G-actin state, A3 may have partially flattened at the time of contact so that the actual docking contact may fall between the two described extremes. Ordering of the A3 D-loop also requires the dynamic opening of the A1 W-loop gate to allow A3 Met^44^ binding (Fig. 4, B and C). The cumulative conformational rearrangements required in both monomers help explain the inherently slow kinetics of actin dimerization (*4*).

A third G-actin subunit, A2, then joins the dimer (Fig. 6A and movie S6, step 2) to complete the trimeric actin nucleus. A2 remains in the G-actin conformation and interacts via its H-loop, which stabilizes the F-actin–like conformation of A3 subdomain 2 (Fig. 5, B and C, and movie S5A). The contact surface between A2 and the A1/A3 dimer is relatively small, consistent with the fast dissociation of this complex (*4*).

### Barbed-end elongation

**Fig. 7.**
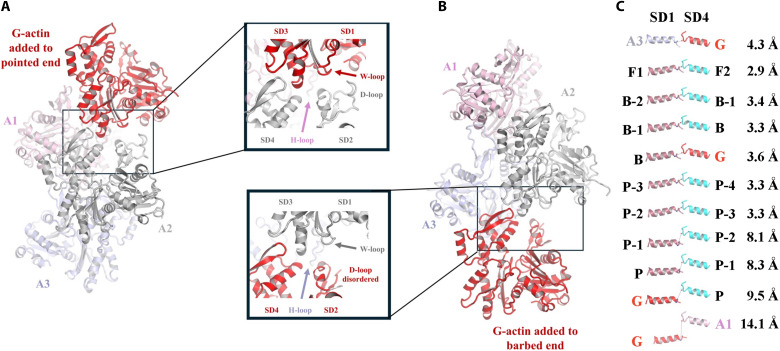
Models of G-actin addition to the pointed and barbed ends of the actin nucleus. The models shown were created by placing G-actin (*17*) onto the actin subunits from the actin nucleus through superimposition onto F-actin via SD3 and SD4 (*25*). (**A**) Nucleus pointed-end model. The A1 H-loop (pink arrow, enlargement) is suitably situated to promote the flattening transition in A2 (gray). (**B**) Nucleus barbed-end model. The A3 H-loop (slate blue arrow, enlargement) is suitably situated to promote the flattening transition in the G-actin subunit (red). No clashes between the main chains of the docked G-actin are observed in either model, nor are there any close contacts between side chains, which cannot be resolved by adjusting side-chain rotamers. Note that the A2 D-loop is ordered because of the crystal contact (fig. S4A); in solution, it will be highly mobile, as observed in the MD simulation (movie S4). (**C**) The helix contact zone between laterally associated actin subunits. Distances are indicated from the main-chain nitrogen of Lys^113^ from the SD1 helix (residues 110 to 128), which is mobile in the flattening transition, to the carbonyl oxygen of Glu^195^ from the relatively static SD4 helix (residues 183 to 198). Distances below 4 Å indicate engaged helices because of the H-bond. B, B-1, and B-2 and P, P-1, P-2, P-3, and P-4 indicate helices from sequential actin subunits from the filament barbed or pointed ends, respectively (*25*). F1 and F2 indicate two subunits from F-actin (*1*). G indicates docked G-actin subunits onto A2 at the pointed end (A) or the barbed end (B). Barbed-end docking (A3 to G) but not pointed-end docking (G to A1) can form an engaged lateral helix zone. Animated versions of this figure are shown in movies S7, S10, and S11, which show G-actin docking and the lateral contacts.

G-actin monomers can associate with either end of the actin nucleus; however, barbed-end addition is likely to be favored because of the more extensive binding site for docking (Fig. 7, fig. S15A, and movie S7). G-actin encounters barbed-end subunits B (A3) and B-1 (A2) (Fig. 6A and movie S6, step 3). In the “SD4 first” scenario (fig. S15B), B-1 SD3 and the B H-loop are positioned to facilitate the docking of the incoming G-actin SD4 (Fig. 7B and movies S7 and S8A). This arrangement, combined with the dynamic opening of the W-loop gate, provides the binding site that promotes the G-to-F flattening transition in SD2 (Fig. 6A and movies S6, step 4, and S8A). The transition is aided by the B subunit SD1 helix, which is correctly positioned because of flattening to form the second type of cross-filament contact with the SD4 helix of the incoming subunit (Fig. 3A, fig. S6B, and movies S7 and S8A). In the “D-loop first” scenario (fig. S15B), after the dynamic opening of the B-1 W-loop, the incoming G-actin D-loop docks, followed by a swing of its SD3 and SD4 to form longitudinal and lateral contacts, including a lateral helix contact with subunit B (Fig. 7C and movies S7 and S8B). Further addition of G-actin to the actin tetramer, barbed-end elongation (Fig. 6A and movie S6, steps 5 and 6), will be facilitated by the lateral helix contact and an open W-loop gate, predicted by the cryo-EM structure of the actin filament barbed end (movie S8, A and B) (*28*). Thus, in the initial phases of polymerization (Fig. 6A and movie S6, steps 1, 2, 4, and 6), subunit addition to the barbed end will sequentially result in the A3 actin being stabilized by one, two, three, and four actin subunits, respectively, implying the increasing stability of the nucleation core, after which sustained polymerization can proceed in a generic manner.

### Pointed-end elongation

Pointed-end filament elongation conceivably proceeds “SD4 first” as the incoming G-actin SD3 binds to the SD4 of the P-1 subunit (asterisk, Fig. 6B) and the H-loop of the pointed-end subunit (Fig. 6B, steps 1 and 3; Fig. 7A; movie S7 and fig. S15, A and C). Alternatively, the incoming G-actin SD3 may bind “H-loop first” to the SD2 and the H-loop of the P-1 subunit (fig. S15C). Either initial contact would trigger the G-to-F flattening transition in the P-2 subunit (Fig. 6B, steps 2 and 4, and movies S6, steps 7 to 9, and S9, A and B) in a transition complex containing three noncanonical F-actin protomer conformations (Fig. 6B, step 1). By contrast, barbed-end addition involves only a single non–F-actin–like subunit in the transition complex (Fig. 6A, step 5) (*31*). The mechanism predicts that addition of two subunits to the pointed end of the original three-subunit nucleus is required to fully integrate the actin nucleus into a filament, with the A1 actin subunit surrounded by four actin subunits (Fig. 6B and movie S6).

### Elongation rates

Filament ends are not equivalent. The pointed end adopts two twisted G-actin–like conformations, whereas the barbed end has two flattened F-actin–like conformations (*25*). This asymmetry has led to several explanations for the slower elongation rate at the pointed end (*6*), including priming of pointed-end subunits for dissociation (*25*), hindrance of the P-2 subunit conformational change (*31*), and partial sequestration of the P-subunit H-loop by the P-1 D-loop (*34*). Considering our proposed activation mechanism, we suggest an explanation based on the docking geometries of the incoming monomers.

At the barbed end, the incoming monomer engages a docking site with three lateral zone interactions with the B subunit (Fig 5C; H1, H2, and lateral helix zone, asterisk in movie S10). At the pointed end, the lateral helix zone is offset, leaving only two lateral interactions for the incoming G-actin with the P subunit (Fig. 7C; H4 and H5 in movie S11). We suggest that this structural asymmetry underlies differential docking (SD4 first) or transition (D-loop first) rates. The more extensive barbed-end site, because of the lateral helix zone interaction, favors faster association and elongation.

Subunit fluctuations may further modulate this asymmetry. W-loop gating in the incoming monomer will slow pointed-end addition (Fig. 4, B, C, and F, and movie S9, A and B), and differences in local flexibility at filament ends may also contribute. Cryo-EM density maps show weaker density for terminal subunits compared to internal subunits (fig. S16, A and B), consistent with high mobility (*25*, *28*, *31*). Subunits B and B-1 form one and two lateral helix contacts, respectively, whereas P and P-1 have none, and P-2 and P-3 form one and two lateral helix contacts, respectively (Fig. 7C and movies S10 and S11). We suggest that B and B-1 are more likely to sample favorable relative geometries for subunit addition because of their stabilizing lateral helix zone contacts, whereas the absence of such contacts in P and P-1 may contribute to slow elongation at the pointed end.

### Monomer docking orientations

The two docking orientations are consistent with the current structural data (fig. S15, B and C). At the barbed end, the “D-loop first” mechanism involves local rearrangements, including the opening of the W-loop gate in the B-1 subunit and ordering of the D-loop in the incoming monomer, to form a compact initial interface with extensive buried surface area. This model aligns with formin INF2 (inverted formin 2)–assisted elongation, where D-loop engagement accompanies the swinging motion that supports processive growth (*35*). MD simulations indicate that longitudinal contacts dominate over lateral ones (*34*), and cryo-EM data showing an open B-1W-loop gate further support this orientation (*28*). Alternatively, the “SD4 first” model proposes a rigid-body mode of addition, in which the incoming monomer docks onto an extended surface formed by the B and B-1 subunits, followed by template-induced flattening. This pathway circumvents the need for major conformational rearrangements during docking, potentially offering a kinetically favorable initial binding. This model is consistent with weaker density for D-loops than for lateral contacts in cryo-EM structures (fig. S16, C to G). At the pointed end, subunit addition is further complicated by the dynamic W-loop gate of the incoming actin monomer. A “D-loop first” pathway would resemble nucleus dimer formation, requiring an open W-loop in the incoming subunit but proceeding more rapidly because of the lateral contacts that promote flattening in P-1. By contrast, an “SD4 first” mechanism would involve the docking of the incoming subunit as a relatively rigid body onto the P and P-1 subunits, followed by W-loop opening and template-induced flattening of P-1. It is plausible that actin uses a combination of geometries depending on local conditions, such as ends or binding partners. Further high-resolution structural and MD analyses will be required to determine whether elongation proceeds through one mechanism or a combination of docking modes.

### Depolymerization

We propose that depolymerization proceeds via the reverse of the elongation mechanisms. At the barbed end, the loss of lateral contacts because of conformational dynamics (*34*) disengages the SD4 lateral helix of the terminal subunit, followed by the release of the D-loop and a switch to the G-actin conformation, facilitating the dissociation of the terminal subunit, which contacts two neighbors (Fig. 6A, steps 5 and 6 in reverse, and movie S6). By contrast, at the pointed end, which also undergoes conformational dynamics, dissociation requires the transient disruption of the P-2 subunit D-loop interaction with the terminal subunit (Fig. 6A, steps 3 and 4 in reverse, and movie S6). The P-2 actin is in the F-conformation and is surrounded by four actin subunits, with the P-3 SD4 helix to P-2 SD1 helix lateral contact restricting the F-to-G twisting transition of P-2 (Fig. 6B, step 4 in reverse, and movie S6; Fig. 7C and movie S11). This constraint impedes the disengagement of the P-2 D-loop from the terminal subunit, slowing release compared to the barbed end (Fig. 6B, step 3 in reverse, and movie S6) (*29*). In the final stages, depolymerization of the trimer involves the dissociation of the laterally positioned subunit, which has a small contact area (movie S4), followed by dimer dissociation and a full reversion to G-actin conformations (movie S6).

In summary, terminal actin subunits are metastable and prone to dissociate unless they are stabilized by additional lateral and longitudinal contacts formed during subunit addition. At high monomer concentrations, association outpaces dissociation and elongation proceeds, particularly at the barbed end, where incoming subunits are incorporated more rapidly because they form a larger set of lateral interactions. At low monomer concentrations, association slows, and dissociation dominates. The pointed end is comparatively more resistant because its conformational transitioning subunit (P-2) makes more lateral and longitudinal filament contacts than the corresponding subunit at the barbed end (B), leaving the barbed end more labile and prone to dissociation (fig. S6B).

### Severing and capping

**Fig. 8.**
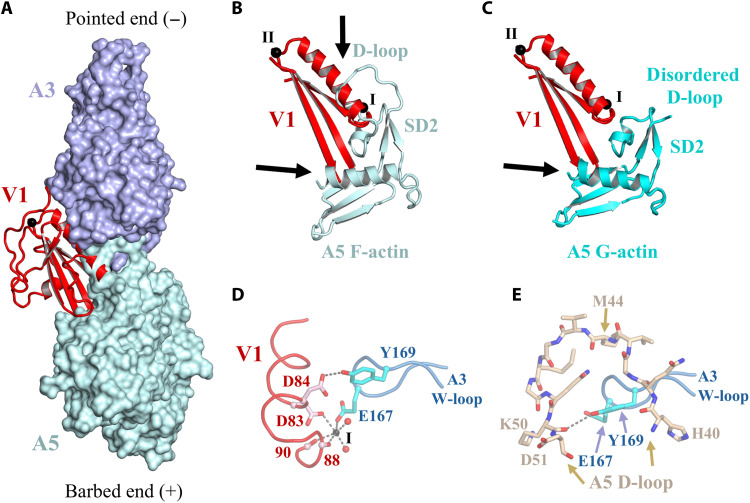
Steric clashes leading to actin filament severing by villin. (**A**) Superimposition of V1 onto two longitudinally related subunits of F-actin (A3 and A5) (*25*). Major steric clashes are seen between the V1 long helix and A5 SD2. (**B** and **C**) Cartoon representations of the clashing region showing that the steric hindrance with the D-loop is lost when A5 is replaced by G-actin. Black arrows indicate steric clashes. Similar steric clashes are seen for V4 (fig. S17). (**D**) In the villin:actin complex, A3 SD3 W-loop residues Glu^167^ and Tyr^169^ respectively coordinate the type I calcium and Asp^84^ of villin. (**E**) In F-actin, A3 SD3 W-loop residue Tyr^169^ coordinates the A5 carbonyl oxygen from D-loop residue 50. Movies S12 and S13 provide animated versions of (A) to (C).

We subsequently turned our attention to the mechanisms by which villin severs and caps actin filaments. Given that A3 adopts an F-actin conformation, villin can now be accurately positioned onto F-actin to give an updated model of actin severing (*12*, *14*). Superimposing A3 from VA3 onto two longitudinally related subunits of an actin filament provides insights into how V1 competes with actin-actin interactions (Fig. 8, A and B, and movie S12). Notably, V1 and the D-loop from the lower actin subunit (A5) bind to the same site on A3, indicating that A5 cannot interact with A3 when V1 is bound. This illustrates V1’s role in filament capping. Conversely, for V1 to access its binding site on the side of a filament, the A5 D-loop must dissociate from A3, allowing A5 to adopt a conformation similar to G-actin (Fig. 8C and movie S13). The long helix of V1 binds to the A3 SD3 (residues 166 to 177, W-loop) through the type I calcium ion that sequesters A3 residue Glu^167^, while V1 Asp^84^ forms a hydrogen bond with A3 Tyr^169^; these interactions fix the position of the W-loop (Fig. 8D). A3 Glu^167^ and Tyr^169^ participate in actin-actin interactions in the context of F-actin and are likely to contribute to the movement in the W-loop, which is required for expanding the Met^44^ binding pocket in F-actin (Fig. 8E) (*4*). These residues are also implicated in the G-to-F flattening transition in the VA3 structure (Fig. 5C). Thus, once V1 binds to these residues, the lower actin (A5) adopts a pointed-end conformation, which cannot revert to the F-actin conformation, destabilizing its interaction with A3. We observed similar mutually exclusive binding sites for V4 and F-actin (fig. S17). In the VA3 structure, V1 and V4 engage tightly with actin via their long helices, whereas V2, V3, V6, and the HP domain exhibit relatively loose associations with actin (figs. S7 and S18). The V3V4 and HP linkers show regions of disorder (Fig. 1, A to D), while the V1V2 linker allows for varying orientations of V1 relative to V2V3 (fig. S17F), indicating the flexibility of these linker regions.

**Fig. 9.**
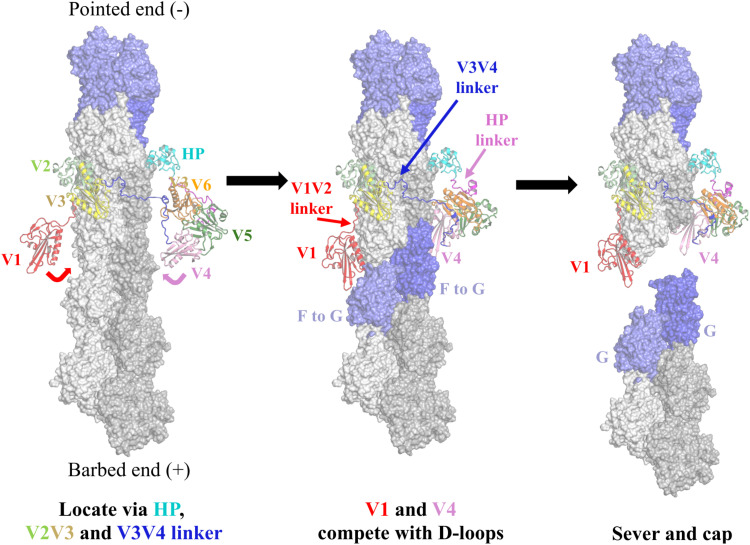
Model of F-actin severing by villin. Initially, V2V3, the V3V4 linker, and the HP domain locate villin to the filament, placing V1 and V4 close to the D-loops of the lower actin subunits. Flexibility in the V1V2, V3V4, and HP linkers allows V1 and V4 to replace the D-loops as they fluctuate between bound and unbound conformations, leaving the lower two actin subunits to adopt G-actin–like pointed-end conformations. Last, the lower severed filament separates from the villin-capped filament. Movie S14 provides an animated version of this figure.

This leads to an updated model of villin-mediated severing of an actin filament (Fig. 9 and movie S14) (*12*). Initially, calcium-activated villin loosely associates with F-actin via the HP domain, V2V3, and the V3V4 linker. The V1V2 linker interacts with the surface of actin, where it positions V1 (and, similarly, the HP linker and V6 position V4) to exploit transient dissociations of the D-loops from the lower actins (movie S14). This D-loop behavior is evident from the weak cryo-EM density of the D-loops compared to the rest of the actin filament in the cryo-EM structure, consistent with partial dissociation (fig. S16D; PDB ID 8A2T) (*1*). By contrast, the electron density is strong for the A3 D-loop in the VA3 map (fig. S16E), indicating that fluctuating D-loop binding is a property of F-actin. Type I calcium ions bind to stabilize the interactions of V1 and V4 with actin. The loss of D-loop interactions destabilizes the F-actin subunit conformation, favoring an F-to-G twisting conformation trajectory, confining the lower actins to pointed-end actin conformations. This destabilizes the filament because of the loss of F-actin interactions (movie S15), resulting in filament severing with the upper fragment capped by villin and the lower fragment capped with pointed-end subunit conformations. This opportunistic mechanism, which uses the transient dissociation of the D-loop, is consistent with the severing activities of a truncation of gelsolin comprising G1 and the G1-G2 linker (*36*) and of the Asgard two-domain gelsolins (comprising G1G2) (*13*, *37*), which are not influenced by the subsequent domains. The cryo-EM structure of a gelsolin-capped filament revealed a SD1 displacement in the G3-bound actin relative to F-actin (*22*), which is also seen, but to a lesser extent, in A3 SD1 from the V3-bound actin (Fig. 2, E and F; Table 1; and movie S3). We suggest that V3-induced SD1 displacement within a filament may increase the D-loop dissociation dynamics and weaken cross-filament interactions by dissociating the helix zone cross-filament contact (movie S16) to allow the multidomain eukaryotic gelsolins and villins to sever more efficiently than their smaller Asgard counterparts.

We propose that villin and gelsolin sever actin filaments by inducing a transition of filament subunits from flat to twisted conformations. This mechanism differs from that suggested by Barrie *et al.* (*22*) who propose that severing is mediated by the rotation of actin subunits within the filament (fig. S5C). In our model, dissociation of the D-loop and the accompanying F-to-G–like twisting transition weaken both longitudinal and lateral filament contacts (movie S15). This mechanism is consistent with observations for other actin-depolymerizing factors: Cryo-EM structures of cofilin-bound filaments show subunit twisting and D-loop dissociation (*38*, *39*), the x-ray structure of twinfilin bound to two G-actin subunits (*40*) supports the idea that twisted conformations and D-loop disengagement promote filament instability, and INF2 has been proposed to disrupt longitudinal D-loop interactions in F-actin during severing on the basis of cryo-EM structures (*35*). We therefore suggest that the reversal of the G-to-F flattening transition and competition for D-loop binding represent common mechanistic principles among actin-depolymerizing factors.

### Implications for villin’s cellular function

Last, we assessed whether the structural insights from the VA3 complex clarify villin’s function, given that the roles of villins in actin dynamics remain to be fully elucidated. The association of villin with a single filament indicates that calcium-activated villin can, like gelsolin, effectively sever and cap individual actin filaments, while its unique HP domain adds additional specificity to this process. This explains why some species, such as plants, do not have gelsolins, only villins—the two proteins are largely interchangeable in their basic functions (*12*). In contrast to gelsolin, mammalian villin’s calcium-free form can bundle actin filaments in vitro at concentrations unlikely to occur in vivo, in line with the observation that bundling is not its primary function (*18*). Instead, we propose that in the cell, inactive mammalian villin preferentially locates to existing or growing actin bundles and acts as a dismantling agent upon calcium signaling, distinguishing itself from gelsolin by acting as a severing agent favoring bundled actin architectures.

## MATERIALS AND METHODS

### Animal collection and experimental design

*P. sul* worms were collected from the Main Endeavour field located along the Juan de Fuca Ridge (47°57′N, 129°5′W) during an expedition on board the *R/V Atlantis* (voyage AT 15-67) in July 2010. All worms were collected from sulfide edifices by the human-occupied vehicle *Alvin* on dive nos. 4409 to 4423, from depths between 2000 and 2200 m, using a multichamber suction sampler or a sampling scoop. Upon collection, worms were placed into a thermally insulated container (*41*). Upon recovery, worms were placed into 20-liter buckets filled with 0.2-μm filter–sterilized seawater and taken into a 4°C cold room where they were visually sorted on the basis of segment number and gill morphology. Only active worms were kept for further study. All mucus and minerals were removed from the remaining animals. All animals were then flash-frozen in liquid nitrogen and stored at −80°C for shoreside laboratory analyses.

### Extraction and reverse transcription

*P. sul* gill tissue (50 to 100 mg) was excised without thawing. Total RNA was isolated from these subsamples using TRIzol RT (MRC Gene, Cincinnati, OH) in a modified single-step RNA extraction method (*41*, *42*). Genomic DNA was removed using TURBO DNA-free reagents (Ambion, Austin, TX), and the resulting RNA was assessed for DNA contamination using the Qubit dsDNA HS Assay (Invitrogen, Carlsbad, CA). All worm RNA extractions had DNA concentrations below the limits of detection (10 pg/μl). The treated RNA product was normalized to 1.75 μg and used as the template in a reverse transcription reaction using random hexamers with the SuperScript VILO cDNA synthesis kit (Invitrogen, Carlsbad, CA).

### Library construction for mRNA sequencing

Samples were quantified using a NanoDrop (Invitrogen) and quality controlled on a 6000 Nano Bioanalyzer chip (Agilent). mRNA enrichment was achieved by processing 6 μg of total RNA using the Magnetic mRNA Isolation Kit from NEB (S1550S). Generation of double-stranded cDNA and library construction were performed using NEBNext mRNA Sample Prep Reagent Set 1 (E6100L) according to the manufacturer’s specifications. Upon ligation of Illumina Adapters (Multiplexing Sample Preparation Oligonucleotide Kit), each library was size selected using a 2% E-Gel EX (Invitrogen). The following custom primers (25 μM each) were used for the polymerase chain reaction (PCR) enrichment step: multiplex PCR primer 1.0, 5′-AATGATACGGCGACCACCGAGATCTACACTCTTTCCCTACACGACGCTCTTCCGATCT-3′; index primer, 5′-CAAGCAGAAGACGGCATACGAGAT[INDEX]CAGTGACTGGAGTTCAGACGTGTGCTCTTCCGATCT-3′.

Indices were according to the eight bases tags developed by the Wellcome Trust Centre for Human Genetics (*43*). The amplified library was purified using Ampure beads (Beckman/Agencourt), and the size distribution was determined using the Agilent DNA 1000 Kit and a 2100 Bioanalyzer Instrument system (Agilent). Libraries were quantified by real-time PCR using the Agilent QPCR Library Quantification Kit and an MX3005P instrument (Agilent) and pooled accordingly. Last, a second real-time PCR was performed to measure the relative concentration of the pool compared to a previously sequenced mRNA library to determine the volume to use for sequencing. Sequencing was performed as a 50–base pair single read on a GAIIx according to Illumina specifications using Cluster Generation Kit version 4 and Sequencing Kit version 4. Oligonucleotide sequences are copyright of Illumina Inc. (2007 to 2011).

### Duplex-specific thermostable nuclease RNA sequencing preparation

Samples were quantified using a NanoDrop (Invitrogen) and quality controlled on a 6000 Nano Bioanalyzer chip (Agilent). mRNA enrichment was achieved by processing 6 μg of total RNA. Generation of double-stranded cDNA and library construction were performed using NEBNext mRNA Sample Prep Reagent Set 1 (E6100L). The double-stranded cDNA library was denatured at 98°C for 2 min and incubated at 68°C for 5 hours. Incubation with the duplex-specific thermostable nuclease enzyme (Evrogen, EA001) was performed as per Illumina’s recommendations. After purification, the library was enriched and sequenced on Illumina machines (*43*).

### Assembly

The full-length *P. sul* transcriptome was assembled using Trinity (*44*) with default parameters. We identified the villin sequence by homology to the human villin sequence (fig. S1).

### Protein expression and purification

The coding sequence for full-length *P. sul* villin (fig. S1) was optimized for expression in *Escherichia coli*, synthesized by GenScript, and subsequently cloned into the pSY5 vector (*45*). This vector incorporates an N-terminal octahistidine tag followed by a cleavage site for human rhinovirus 3C protease. A truncated version of *P. sul* villin, V4V6-HP (residues 355 to 824; which includes the V3V4 linker), was introduced into the pSY5 expression vector using PCR cloning and Gibson assembly techniques.

Overnight cultures were used to inoculate fresh LB media containing ampicillin, which was then shaken at 37°C until the OD_600_ (optical density at 600 nm) reached ~0.6. Protein expression was induced with 0.25 mM isopropyl β-d-1-thiogalactopyranoside and allowed to proceed overnight at 16°C. Following induction, cells were harvested by centrifugation at 4000*g* for 1 hour at 4°C, and the resulting pellets were resuspended in 50 ml of His-binding buffer (20 mM tris-HCl, pH 7.5, 500 mM NaCl, and 20 mM imidazole) supplemented with an EDTA-free protease inhibitor cocktail (Calbiochem) and 2 μl of benzonase (10,000 U/μl; Merck). Lysis was achieved using an ultrasonic cell disruptor (Branson) with a pulse duration of 5 s and a duty cycle of 30 to 40% for 5 min. The lysate was then applied to a Ni-NTA affinity chromatography column (Qiagen) and washed with five column volumes of binding buffer. The N-terminal octahistidine tag was cleaved using HRV-3C protease at 4°C overnight. The eluted proteins in the binding buffer were further purified by size exclusion chromatography (Enrich SEC 70, Bio-Rad) using a buffer composed of 20 mM tris-HCl, pH 7.5, and 150 mM NaCl. Last, the proteins were concentrated using 10-kDa-MWCO (molecular weight cutoff) centrifuge filters (Merck).

### Preparation of the *P. sul* villin:actin complexes

ATP-G-actin was purified from chicken skeletal muscle acetone powder (*24*) and subjected to size exclusion chromatography. The purity of G-actin was evaluated via SDS–polyacrylamide gel electrophoresis, and its concentration was determined by measuring the absorbance at 290 nm. ADP-actin was prepared by polymerizing ATP-G-actin in 0.2 M KCl, 20 mM imidazole, pH 7.0, 2 mM MgCl_2_, 0.4 mM EGTA, 0.4 mM ADP, 2 mM dithiothreitol (DTT), 10 mM glucose, and hexokinase (15 U/ml ). After 2 hours, the solution was centrifuged (100,000*g* for 30 min), and the pellet was homogenized in 2 mM tris-HCl, pH 8.0, 0.2 mM CaCl_2_, 0.2 mM ADP, 1.0 mM DTT, 10 mM glucose, and hexokinase (15 U/ml) and kept on ice for 2 hours before being centrifuged (100,000*g* for 30 min). The supernatant contained ADP-G-actin.

The VA3 protein complex was formed by combining *P. sul* villin and chicken ADP-G-actin in 2 mM tris-HCl, pH 8.0, 0.2 mM ADP, 1.0 mM DTT, and 1 mM CaCl_2_ or chicken ATP-G-actin in 2 mM tris-HCl, pH 8.0, 0.2 mM ATP, 1.0 mM DTT, and 1 mM CaCl_2_ at a molar ratios of 1:3. The mixtures were incubated on ice for 10 min to facilitate complex formation and then purified by size exclusion chromatography in the same buffers. The V1V3/actin crystals resulted from full-length villin mixed at a 1:2 ratio with ATP-G-actin without a subsequent gel filtration step in buffer A (2 mM tris-HCl, pH 7, 0.2 mM ATP, 0.5 mM DTT, 1 mM NaN_3_, and 0.1 mM CaCl_2_). The V4V6-HP/actin complex was made by mixing V4V6-HP with ATP-G-actin (1:1 ratio) followed by gel filtration in buffer A.

### Crystallization

Complexes were concentrated using a Vivaspin 20 MWCO 10,000 concentrator (Sartorius) to ~10 mg/ml. Protein crystallization experiments were conducted via the sitting-drop method using commercial screening kits in 96-well Violamo crystallization plates at room temperature (20°C). Initially, the VA3 complex was formed using ATP-G-actin to identify the VA3 crystallization condition. Inspection of the electron density maps resulting from these crystals revealed ADP bound to all three actin subunits. The final VA3 crystals were formed using ADP-G-actin, which extended the resolution by ~0.5 Å. The optimized conditions for crystallization were as follows: for VA3, 0.1 M bis-tris propane, pH 6.5, 10% polyethylene glycol, molecular weight 3350 (PEG 3350), 0.2 M KSCN, and 5 mM CaCl_2_/MgCl_2_; for V1V3/actin, 0.1 M bis-tris propane, pH 8.5, 20% PEG 3350, 0.2 M sodium nitrate, and 1 mM CaCl_2_; for V4V6-HP, 0.1 M tris-HCl, pH 8.0, 50% PEG 400, 0.2 M Li_2_SO_4_, and 1 mM CaCl_2_. Crystals of V1V3/actin and V4V6-HP were cryocooled directly in their respective crystallization buffers, while VA3 crystals were supplemented with 14% glycerol before cryocooling for data collection.

### Crystallography data collection and structure determination

Data collection for a single crystal of V1V3/actin was performed using a Rayonix MX300-HS CCD detector at the National Synchrotron Radiation Research Center, Taiwan, ROC, using beamline TPS 05A at a wavelength of λ = 1.0 Å. Indexing, scaling, and merging of the dataset were accomplished using HKL2000 (*46*). Data for VA3 and V4V6-HP crystals were collected at beamline BL41XU (λ = 1.0 Å) at the SPring-8 synchrotron, Japan, using a PILATUS 6M detector, and data processing followed standard protocols (*47*). Molecular replacement and refinement of the VA3 structure used individual domains from G1G3/actin, G4G6/actin, and villin HP structures (PDB IDs 3FFK, 1H1V, and 3NKJ, respectively) (*23*, *45*, *48*). The refined model (9JUS) domains served as molecular replacement models for solving the structures of V1V3/actin and V4V6-HP/actin. All models were refined using PHENIX and rebuilt in COOT (*49*, *50*). Because of the low resolution of the V4V6-HP/actin crystal data (3.29 Å), noncrystallographic torsion-angle restraints were applied throughout refinement. The VA3 crystals contain two VA3 complexes in the asymmetric unit, with each actin subunit associated with Mg-ADP and each villin molecule bound to eight calcium ions. Villin chains, v and V, comprise residues 2 to 824 with disordered regions in the V3V4 linker (v, 360 to 364; V, 361 to 366) and in the HP-linker (752 to 754). V1 showed clearer electron density in chain v relative to chain V. The F-actin A3 subunits comprise residues 4 to 375; the A2 subunits comprise residues 4 to 364, with disordered C-termini (residues 365 to 375); and the A1 subunits comprise residues 4 to 375 with disordered D-loops (chain g, residues 41 to 50; chain G, residues 41 to 51). The V1V3/actin crystals feature a single complex copy, with actin bound to Ca-ATP and V1V3 associated with four calcium ions. V1V3 consists of residues 2 to 353 with no density for the V3V4 linker and actin residues 5 to 39 and 50 to 368, with a disordered D-loop and C terminus. The V4V6-HP/actin crystals consist of two copies of the V4V6-HP/actin complex, with actin bound to Ca-ADP and V4V6 associated with four calcium ions. These crystals exhibit domain swapping of the V3V4 linker between the two molecules in the asymmetric unit. V4V6-HP chain V comprises residues 370 to 750, and the electron density is missing for the HP domain. V4V6-HP chain v comprises residues 376 to 385, 393 to 749, and 757 to 824, with breaks in the V3V4 linker and HP linker densities. Actin chain g comprises residues 1 to 38, 52 to 59, and 64 to 369, and chain G comprises residues 3 to 38 and 52 to 374, both having disordered D-loops but showing variability in the ordered state of the C terminus.

### Actin subunit analysis

Center-of-mass calculations were carried out on the basis of the following residues from each subdomain in PyMOL (*51*): SD1 7 to 31, 74 to 136, and 347 to 364; SD2 34 to 39 and 53 to 68; SD3 149 to 179 and 274 to 331; SD4 185 to 256. These residues were chosen because they are present in each of the structures analyzed in Table 1. The dihedral angle, ϕ SD1–SD4, and distances and angles were measured in coot (*49*). Buried surface areas were calculated in PISA (*52*).

### MD simulations

To evaluate the dynamics of the A1, A2, and A3 actin trimer, all-atomistic MD simulations were performed with the GROMACS 2024.2 (*53*, *54*), with the system preparation performed through CHARMM-GUI version 3.7 (*55*–*57*). The D-loop from A2 was grafted onto A1 to complete the chain for the A1 subunit. A2 in isolation was used as the starting conformation for the monomer (Table 1). The system consisted of a solvated actin monomer or trimer protein complex parameterized with the CHARMM36m force field (*58*) and solvated in a cubic TIP3P water box with a minimum buffer of 10 Å from any protein atom to the box edge. The system was neutralized with Na^+^ and Cl^−^ counterions and adjusted to an ionic strength of 0.15 M. Periodic boundary conditions (*59*) were applied in all directions. Energy minimization was performed using the steepest descent algorithm with harmonic position restraints on backbone and side-chain atoms using force constants of 400 and 40 kJ mol^−1^ nm^−2^, respectively. The minimization terminated when the maximum force dropped below 1000 kJ mol^−1^ nm^−1^ or a maximum of 5000 steps was reached. For the equilibration process, this step was conducted in the NVT ensemble at 298.15 K using the stochastic velocity rescaling (v-rescale) thermostat (*60*) with a time constant of 1 ps and separate coupling groups for solute and solvent. A 125-ps simulation was performed using a 1-fs time step. Harmonic restraints on the backbone and side chains were maintained as in the minimization step. The v-rescale thermostat was selected for its ability to maintain temperature stability while ensuring accurate sampling of the canonical ensemble, balancing thermodynamic reliability and numerical stability (*60*).

The production MD simulation was subsequently performed in the NPT ensemble at 298.15 K and 1 bar using the v-rescale thermostat for temperature control and the stochastic cell rescaling (C-rescale) barostat for isotropic pressure coupling (time constant, 5.0 ps; compressibility, 4.5 × 10^−5^ bar^−1^). The C-rescale barostat was selected for its enhanced numerical stability and energy conservation compared to the traditional Berendsen method, making it suitable for long production runs in the isothermal-isobaric ensemble (*61*). A 2-fs integration time step was used for a 1-μs simulation, with conformations and energies recorded every 10 ps. The LINCS algorithm (*62*) was used to constrain all hydrogen-containing bonds. Nonbonded interactions were handled with the Verlet cutoff scheme (*63*), with cutoffs of 12 Å for both van der Waals and electrostatic interactions. Particle mesh Ewald (*64*, *65*) was applied for long-range electrostatics.

## Supplementary Materials

### The PDF file includes:

Figs. S1 to S19 Table S1 Legends for movies S1 to S16 References
