# Supplementary material for: The structure of an actin nucleus stabilized by villin
Source: Sci Adv. 2025 Dec 3;11(49):eadw6915. doi: 10.1126/sciadv.adw6915 (PMC12674126; doi:10.1126/sciadv.adw6915)
Supplement: Supplementary file 1 — Figs. S1 to S19 Table S1 Legends for movies S1 to S16 References [file sciadv.adw6915_sm.pdf]

Science Advances
AAAS

Supplementary Materials for

The structure of an actin nucleus stabilized by villin

Robert C. Robinson *et al.*

Corresponding author: Robert C. Robinson, robert.b@vistec.ac.th

Sci. Adv. **11**, eadw6915 (2025)
DOI: 10.1126/sciadv.adw6915

The PDF file includes:

Figs. S1 to S19
Table S1
Legends for movies S1 to S16
References

Other Supplementary Material for this manuscript includes the following:

Movies S1 to S16

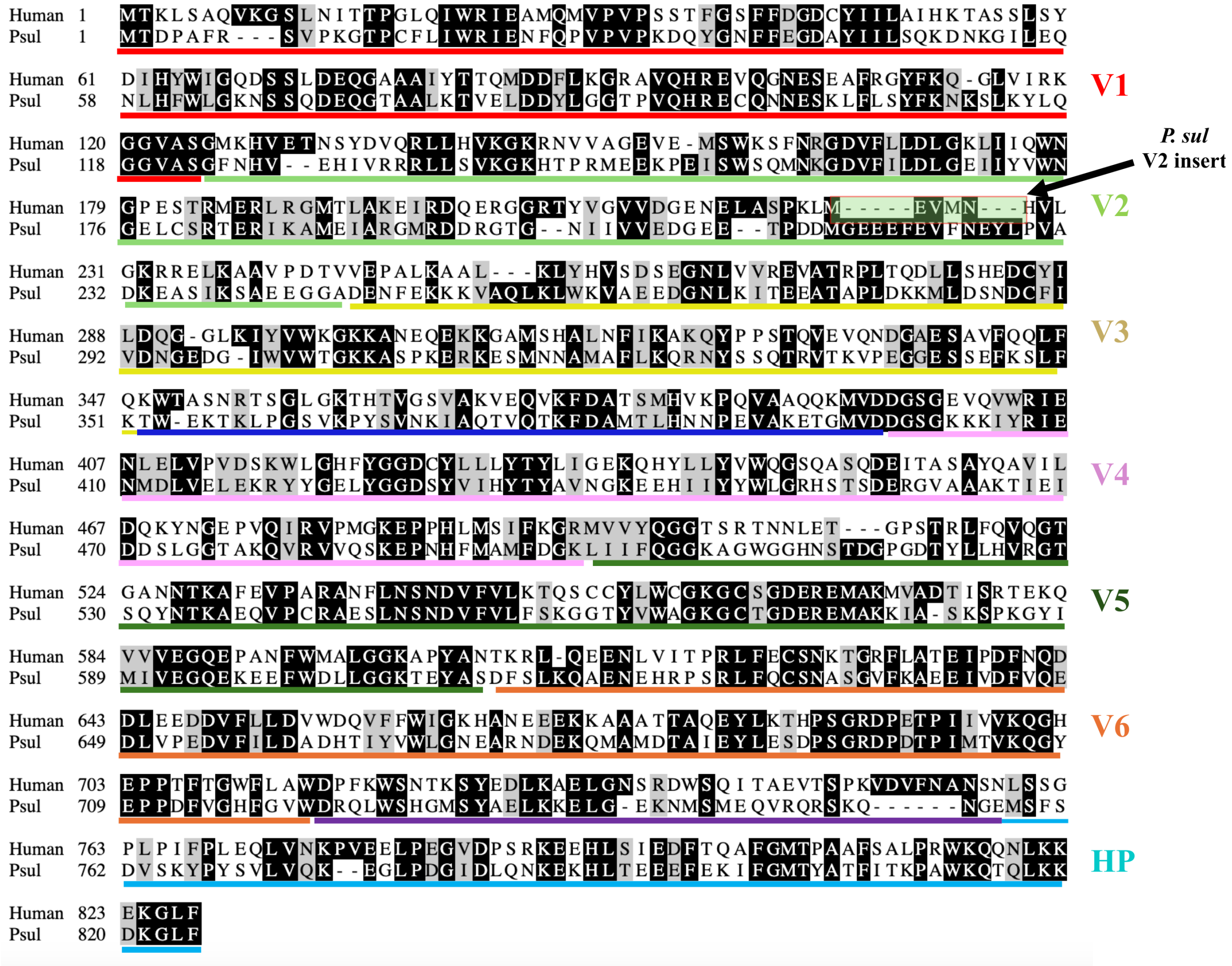

Fig. S1. Alignment of *P. sul* and human villin protein sequences. Colored lines below the alignment indicate the domain structure shown in Fig. 1. Green shading indicates the *P. sul* V2 insert relative to human villin.

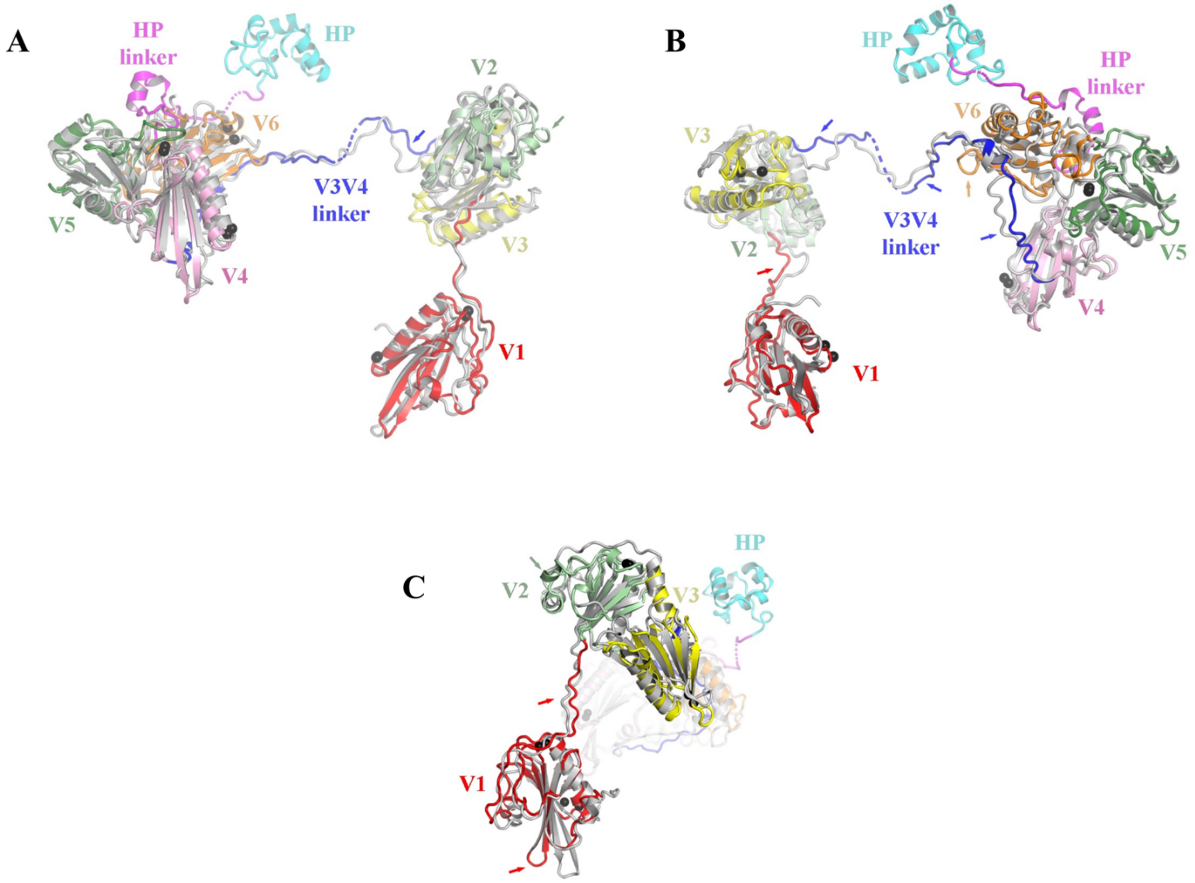

Fig. S2. Superimposition of the calcium and actin bound *P. sul* villin and human gelsolin structures. The villin domains are colored as in Fig. 1, and the actins have been omitted for clarity. Gelsolin, from the cryo-EM structure of the gelsolin-capped actin filament (PDB ID 8VIZ), is shown in gray (*22*). (**A**) View similar to that in Fig. 1C. (**B**) Rotated by ~180 ° around the vertical axis. **C**) View focusing on V1V3. Arrows indicate structural divergence, which often occurs in linker regions. An animated version of this figure is shown in movie S2.

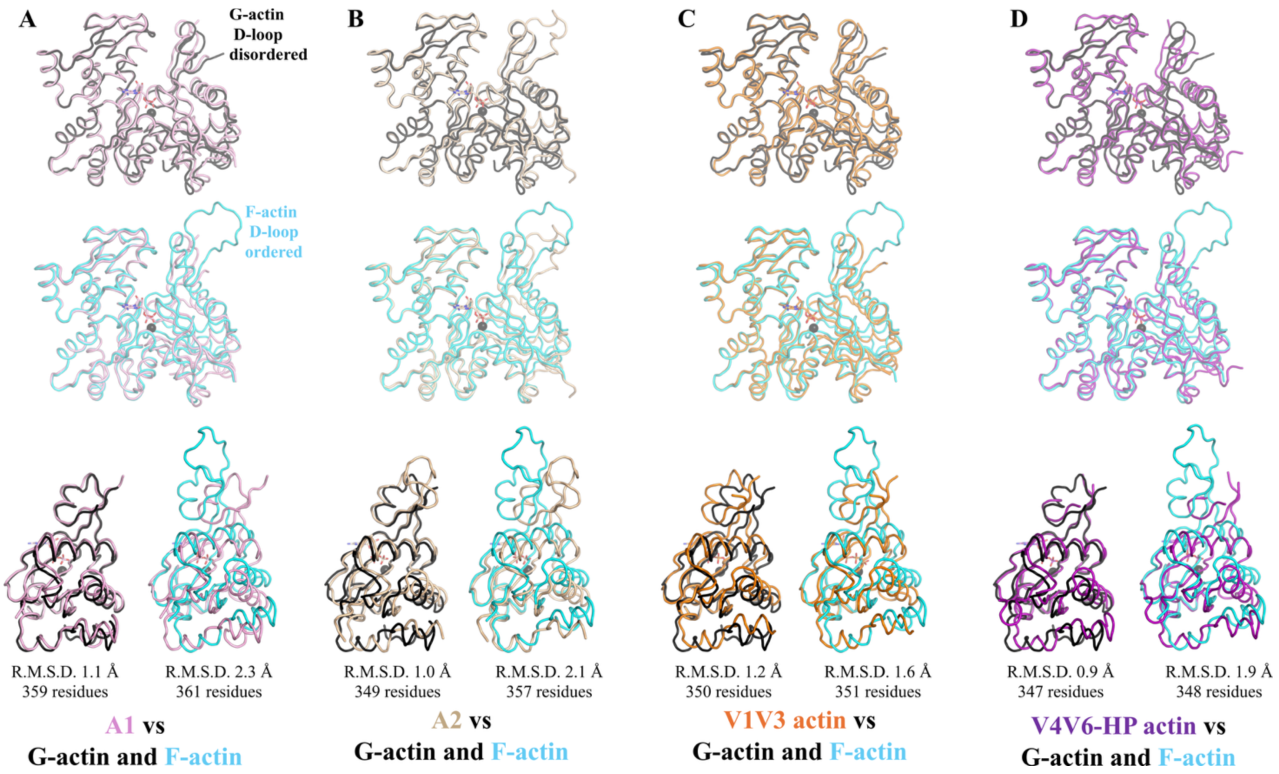

Fig. S3. Comparison of the villin-bound actin conformations with G- and F-actin. Superimpositions onto G-actin (black, PDB ID 3HBT) and F-actin (cyan, PDB ID 8F8S) (*24*, *25*) of A1 (**A**, pink) and A2 (**B**, tan) from the VA3 complex, actin from the V1V3 complex (**C**, orange), and actin from the V4V6-HP complex (**D**, magenta). The actin subunits in (**A**), (**B**), and (**D**) adopt G-actin conformations, while the actin subunit in (**C**) adopts an intermediate conformation.

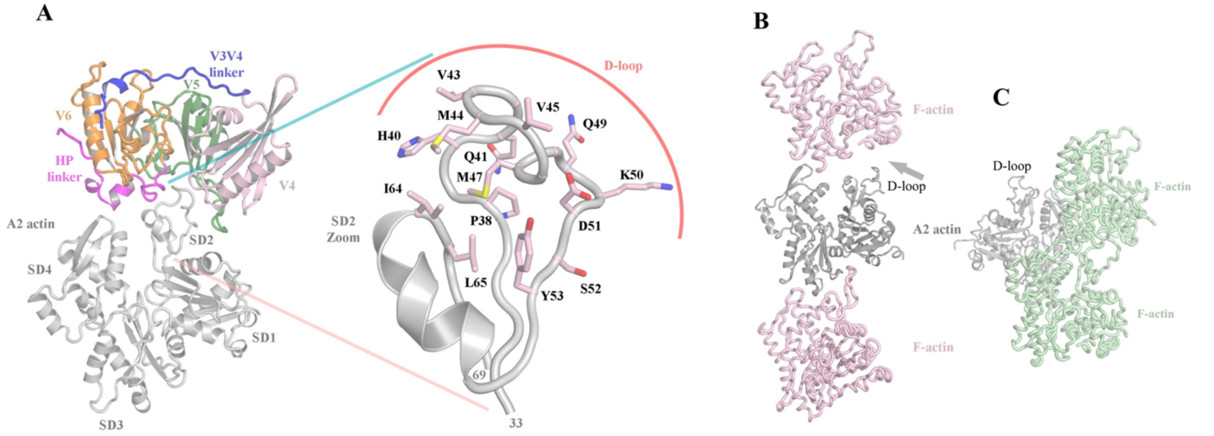

Fig. S4. Crystal contacts stabilize the actin A2 subunit D-loop. (**A**) A2 actin subunit is shown in gray and the V4V6 portion of villin, from the second complex in the crystallographic asymmetric unit, is colored. The zoomed SD2 region reveals that Met47 binds back into a hydrophobic pocket of SD2, enabling the D-loop to adopt a compact structure. The buried area at this crystal contact is 670 Å^2. (**B**) The A2 actin subunit superimposed on one strand of an actin filament. This model indicates that compact D-loop conformation (arrow) is incompatible with forming longitudinal interactions within a protofilament. (**C**) The A2 actin subunit superimposed on the laterally-related strand of an actin filament. This model indicates that the compact D-loop conformation cannot form lateral interactions across a filament. We interpret the A2 compact D-loop conformation to be induced by the crystal packing, and likely the D-loop will be mobile in the absence of the crystal contact. Crystal packing induced D-loop conformations have been observed before (*66*, *67*).

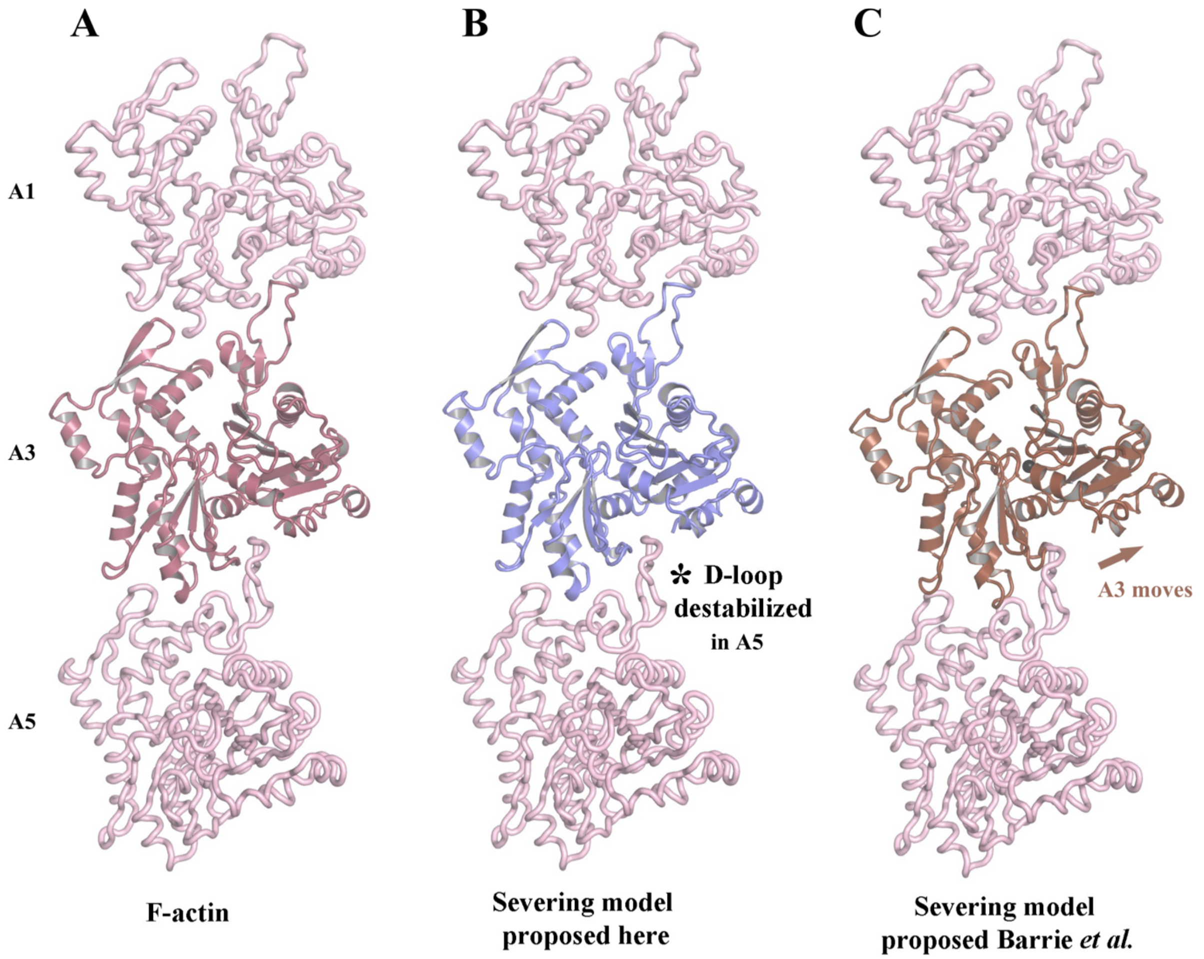

Fig. S5. Superimposition of actin subunits on one strand of an actin filament. (**A**) F-actin (ribbon) with the central protomer shown as cartoon. (**B**) The VA3 A3 actin (light blue) after being superimposed on the central protomer (A3) from **A**. In the mechanism of severing proposed here, villin binding destabilizes the D-loop in A5 to allow V1 to bind to A3. This represents a dissociation event that does not require a major disruption to the filament structure. (**C**) The gelsolin G1 bound actin subunit (brown) orientated after superimposition of the next longitudinally-related actin subunit towards the pointed end, from the cryo-EM structure of the gelsolin-capped filament, onto A1 from **A**. In the mechanism of severing proposed by Barrie et al. (*22*), gelsolin binding causes A3 to rotate from the position in **A** to reveal the gelsolin G1 binding site on A3. This would require a major disruption at the A3/A5 interface as modeled (*22*).

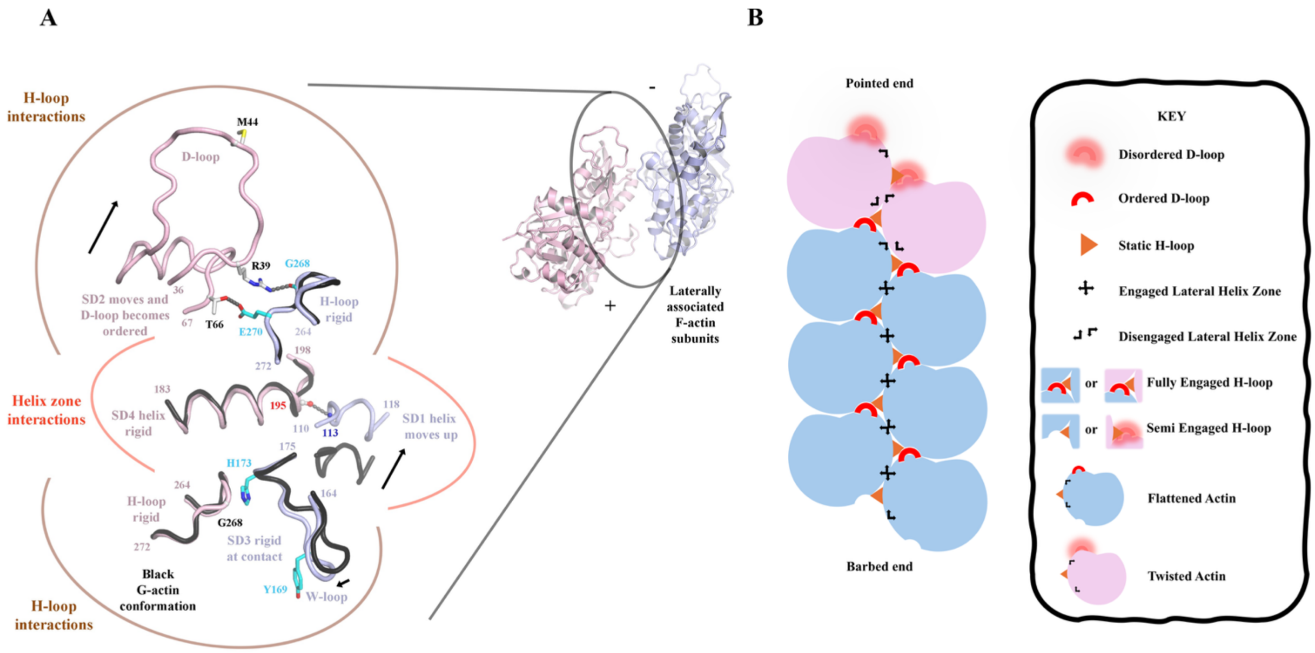

Fig. S6. Lateral contacts across an actin filament. The lateral contacts across the filament are depicted for reference (*26*). In the zoom depiction of the interacting residues, G-actin (black) is superimposed to highlight the G-to-F conformational changes in these regions. However, the G-actin superimposition on the F-actin SD2 and D-loop is omitted for clarity. These details are found in Fig. 5, A and B. (**A**) There are two types of lateral interaction zones:

The H-loop zone, in which the static H-loop forms two H-bonds across the filament with the mobile SD2 (Top: G268:R39 and E270:T66) and forms a Van der Waals interaction with another static region at the base of the W-loop (Bottom: G268:H173). These contacts occur at the junction of three actin subunits. Thus, in the actin filament, each internal subunit forms three H-loop interaction zones: its own H-loop interacting with two lateral subunits (Glu270 and Gly268 with the lower lateral subunit, and Gly268 interacting with His173 from the upper lateral subunit), its D-loop interacting with the upper lateral subunit H-loop via Glu270 and Gly268 (not shown), and its His173 interacting with the lower lateral actin H-loop (movie S16). In VA3, actin A2 residues G268 and E270 interact with residues R39 and T66 from actin A3, while actin A2 residues G268 interacts with actin A1 residue H173 (Fig. 5C). H173 has two alternate conformations in A1 in the VA3 structure, one of which corresponds to the conformation depicted here.

The helix zone, in which residue E195 from the end of the static SD4 helix (residues 183-195) forms a mainchain H-bond to K113 from the beginning of the mobile SD1 helix (112-118). The sidechains of these residues, and surrounding residues (L110 and P112 to K191 and T196, not shown) form Van der Waals interactions around the helices' point of contact. This suggests the static regions provide the template to induce and stabilize the F-actin conformation in the mobile regions. Thus, in the actin filament, each internal subunit forms 2 helix zone interactions: its own SD1 helix interacts with the SD4 helix from the laterally related lower subunit, and its SD4 helix interacting with the SD1 helix from the laterally related upper subunit (not shown) (movie S16).

(**B**) In summary, each internal F-actin subunit is involved in five lateral zone interactions: 3 H-loop zones and 2 helix zones. Whereas contacts between two laterally-related subunits are limited to three interaction zones: 2 H-loop zones and 1 helix zone (movie S16). At the barbed end the B subunit is involved with 1 full and 1 partial H-loop zones and 1 helix zone, whereas at the pointed end the P-1 subunit participates in 2 full H-loop zones and no helix zone, and the P subunit is engaged by 1 full and 1 partial H-loop zones and no helix zone.

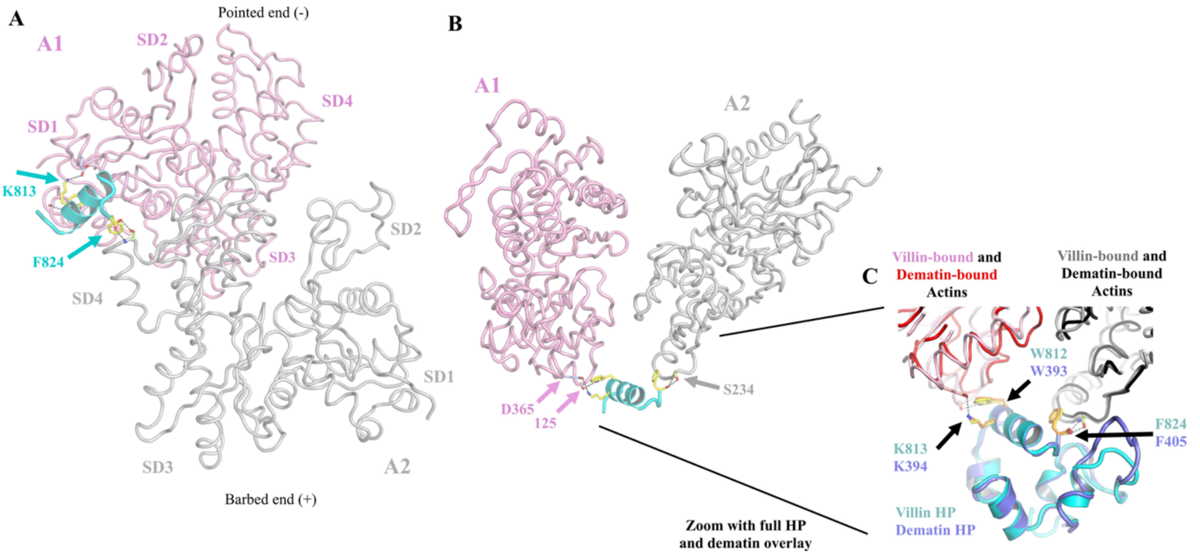

Fig. S7. The C-terminal helix of the villin HP domain. (**A** and **B**) Two views that highlight the C-terminal helix (cyan with yellow residues) binding at the junction of two laterally associated actin subunits (A1 and A2). Contact interactions are labeled. Actin is comprised of four subdomains (SD1-SD4) which are labeled in (**A**). (**C**) Superimposition of the HP domains from villin and dematin, with their associated actin subunits. Both HP domains bind at the junction of two laterally associated actins using conserved residues.

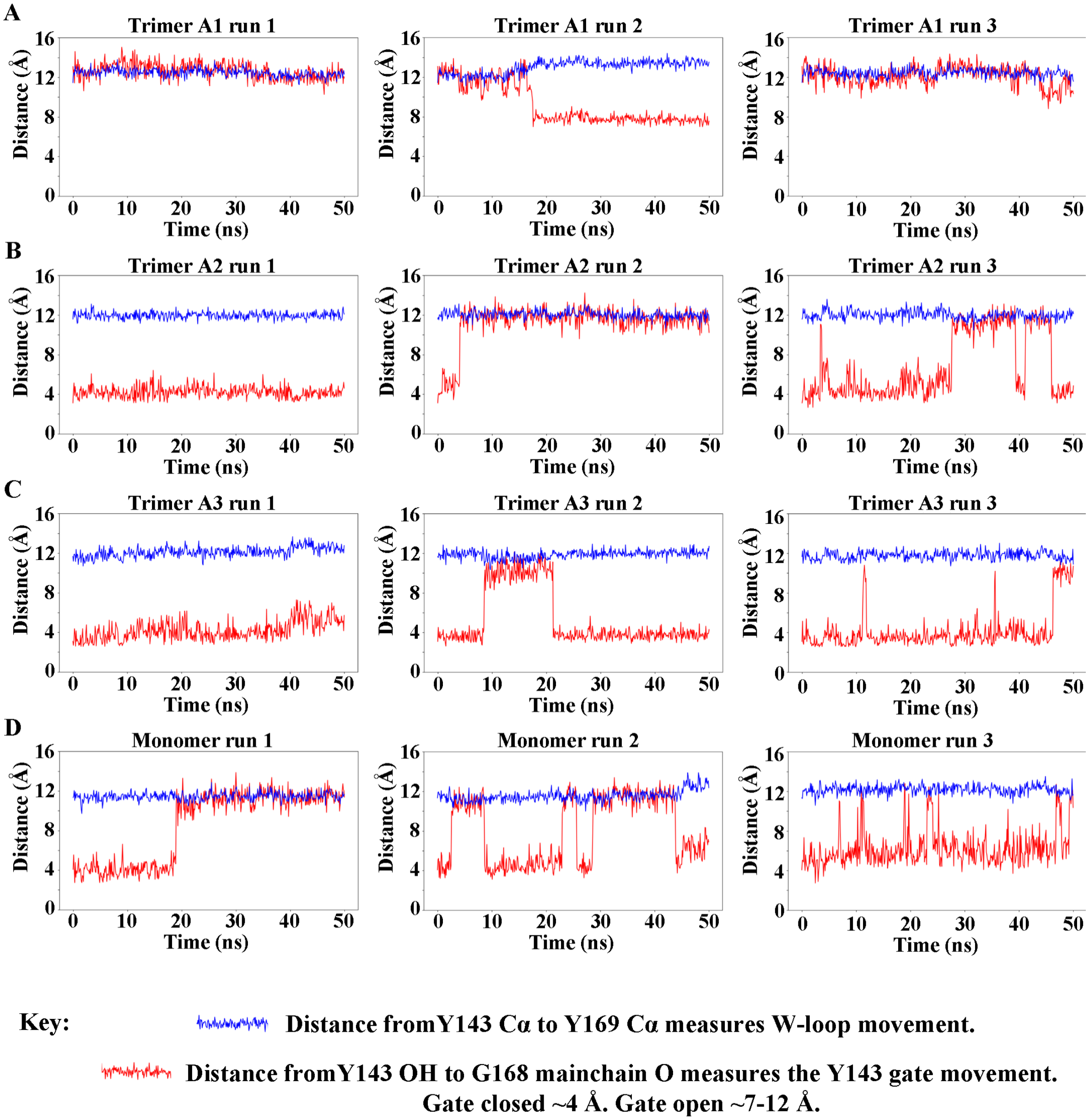

Fig. S8. The W-loop gate for the Met44 binding site. (**A-C**) Three 50 ns MD simulations of the actin trimer. Traces indicate the distances between Tyr143 and Tyr169 Cα atoms (blue) or between the Tyr143 OH to Gly168 O atoms (red). When the OH-O distance is ~ 4 Å the gate is shut and at 8-12 Å the gate is open. (**A**) A1, the gate is open and bound to the A3 D-loop (Fig. 5C). (**B**) A2, the gate opens and closes in two runs. (**C**) A3, the gate opens and closes in two runs. (**D**) G-actin displays opening and closing gate characteristics in all three runs.

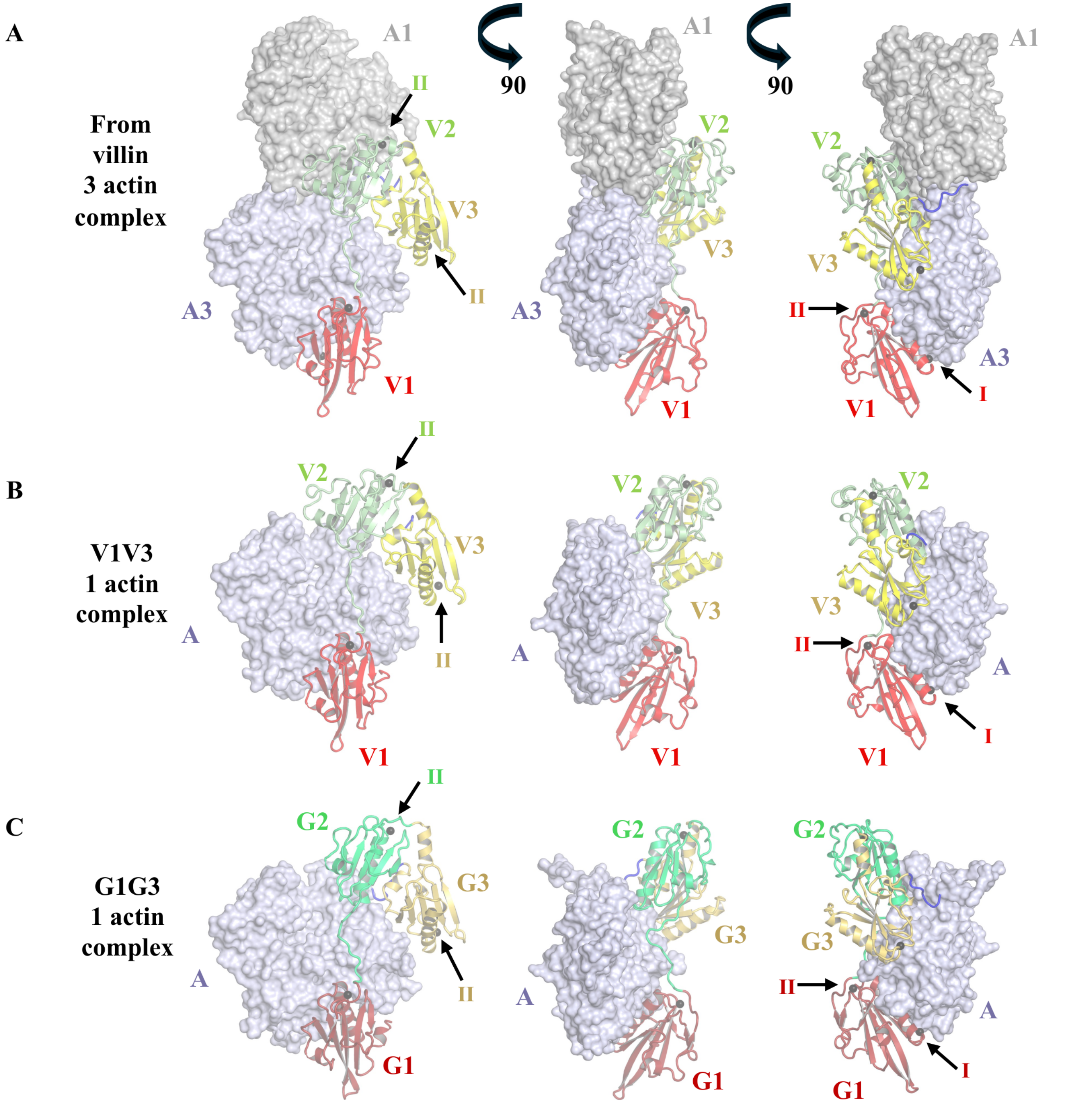

Fig. S9. The interactions of villin N-terminal domains with actin. V1V3 binds to actin similarly to the corresponding region of villin in the VA3 complex and in the X-ray structure of the gelsolin G1G3/actin complex. (**A**) Three views taken from the VA3 structure (Fig. 1A). (**B**) Three views taken from the V1V3:actin structure. (**C**) Three views taken from the X-ray structure of the gelsolin G1G3:actin structure (PDB ID 3FFK) (*45*). In each case, villin or gelsolin domains adopt similar positions on the face of the lower actin (A3 in (**A**)), which are not dramatically changed with the addition of the upper actin (A1 in (**A**)). Each structure features four conserved and occupied calcium ion-binding sites. Three type II and one type I calcium-binding sites are labeled and color-coded to their domains.

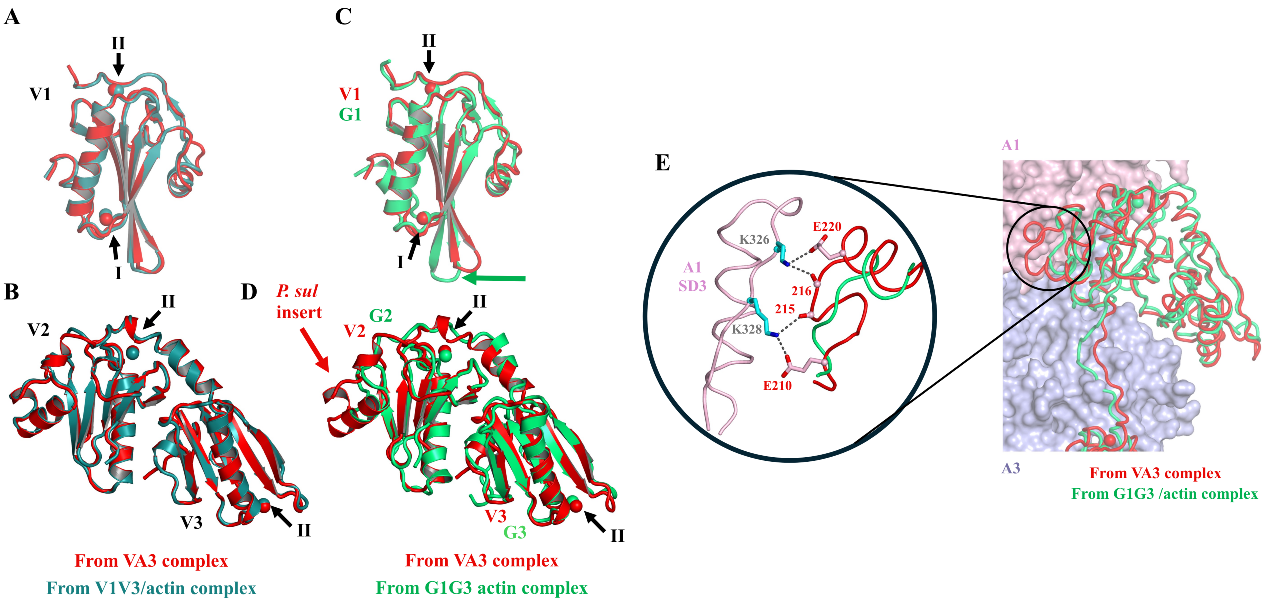

Fig. S10. N-terminal domain structure comparisons. (**A** and **B**) Superimpositions of the (**A**) V1 domains and the (**B**) V2-V3 domains from the VA3 (red) and V1V3:actin (blue) structures, showing their high similarity. Black arrows indicate type I and II calcium ions. (**C** and **D**) Gelsolin N-terminal domains are also highly similar from the X-ray structure of the G1G3/actin complex (PDB ID 3FFK) (*45*). Green arrow indicates a difference in conformation of the extended strand pair in G1 vs. V1; red arrow indicates an insert in *P. sul* V2 that is not present in human gelsolin or villin (fig. S1). (**E**) The *P. sul* insert in V2 forms additional contacts with actin. A1 SD3 residue Lys326 interacts with V2 Glu220 and the carbonyl oxygen of residue 216, and A1 SD3 residue Lys328 interacts with V2 Glu210 and the carbonyl oxygen of residue 215. This interaction may enhance the stability of the complex, facilitating crystallization in our study. The enlargement is rotated relative to the larger view.

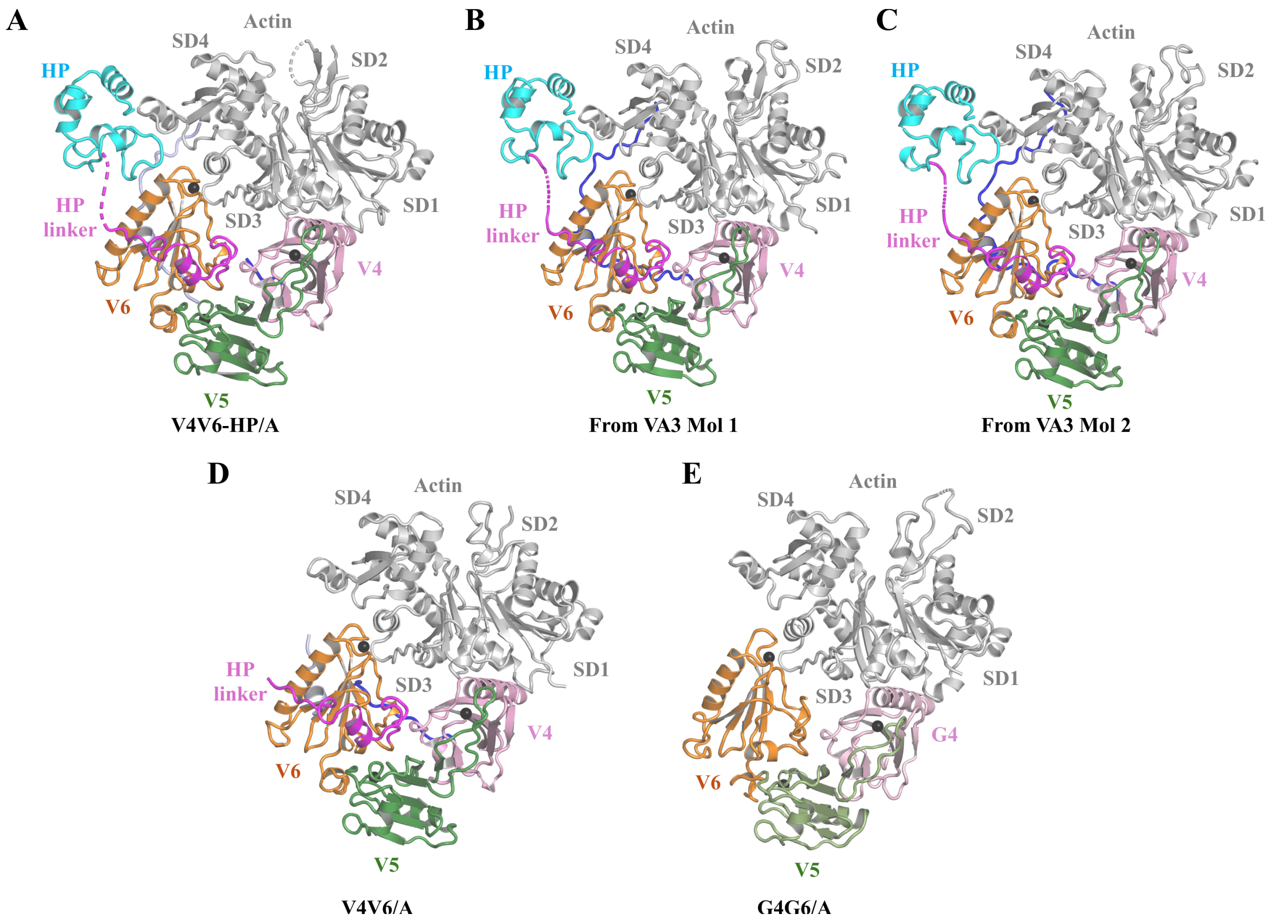

Fig. S11. V4V6-HP/actin structure comparisons. (**A**) The V4V6-HP/actin structure generated from the crystallization of the C-terminal half of villin bound to actin. The villin domains are colored as in Fig. 1. Actin is colored in gray, with the subdomains (SD) labeled. (**B** and **C**) The same protein chains taken from the VA3 structure; (**B**) molecule 1 and (**C**) molecule 2, from the two copies in the VA3 crystal asymmetric unit. The V4V6 domains adopt similar positions in binding actin within the three structures. The HP domain binds to SD4 of actin but is slightly tilted in (**A**) relative to (**B**) and (**C**), where it is stabilized by interactions with a second actin (fig. S7). (**D**) The second molecule in the V4V6-HP/actin crystal lacks an ordered HP domain but is otherwise similar to (**A**). Since the HP domain is slightly tilted relative to actin SD4 in (**A**) and is absent in (**D**), it appears that two laterally related actin subunits are required for stable HP domain binding (Fig. 1). (**E**) The X-ray structure of the gelsolin G4G6 domains bound to actin for comparison (PDB ID 1H1V) (*23*). V4V6 and G4G6 adopt similar positions in binding actin. All structures (**A** to **E**) bind to calcium ions at the one type I and three type II binding sites.

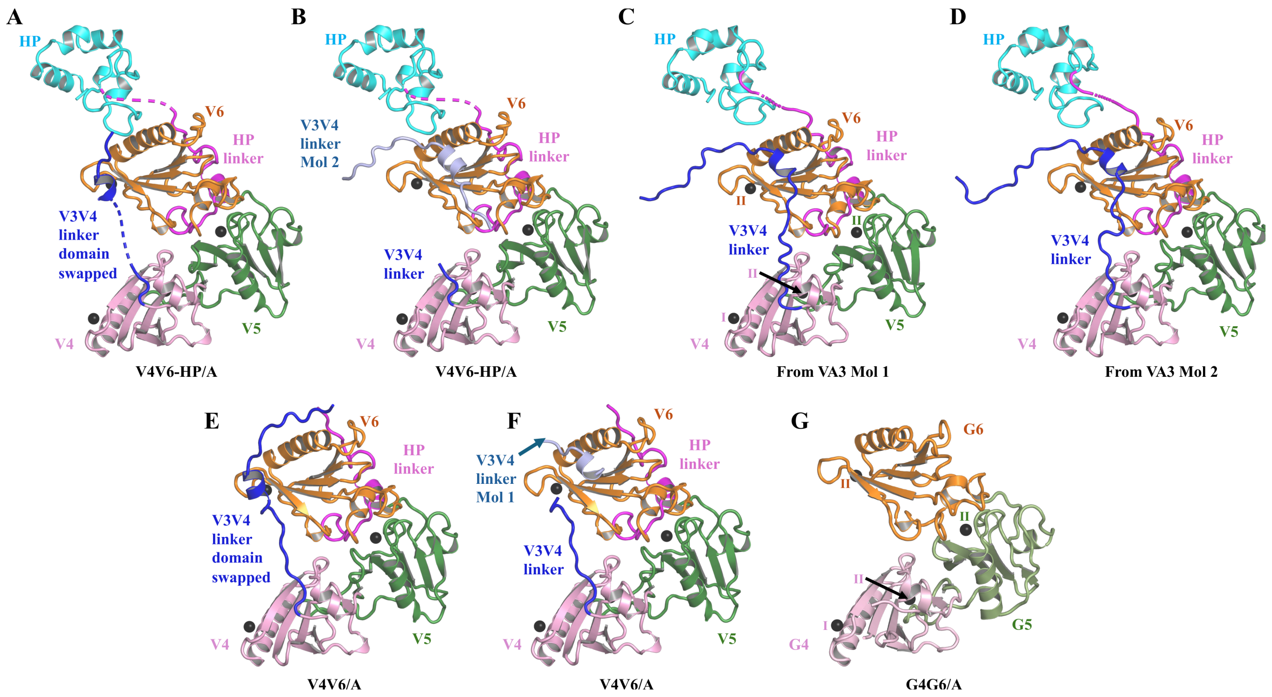

Fig. S12. The V3V4 linker undergoes domain swapping in the V4V6-HP/actin crystals. (**A**) Molecule 1 of the V4V6-HP structure from the crystal of the C-terminal half of villin bound to actin. The bound actin is not shown. V3V4 linker domain swapped, refers to this linker from molecule 1 interacts with molecule 2 rather than with molecule 1. (**B**) Molecule 1 structure, as in (**A**), replacing the molecule 1 V3V4 linker with that from molecule 2 (slate blue) in the asymmetric unit. (**C** and **D**), Comparison of the V3V4 linkers from the two copies of the VA3 complex from the asymmetric unit. The path of the molecule 2 V3V4 linker across V6 in (**B**) is similar to the path of the V3V4 linker in (**C**) and (**D**). (**E**) Similarly, in molecule 2 from the V4V6-HP crystal (V4V6; the HP has no electron density), the V3V4 linker (blue) contacts V6 in a different region than in VA3. (**F**) The same structure in (**E**), replacing the V3V4 linker (slate blue) from molecule 1 in the asymmetric unit, which lies in a similar position on V6 as in the two molecules of the VA3 complex (**C** and **D**). (**G**) The X-ray structure of the gelsolin G4G6 domains for comparison (PDB ID 1H1V) (*23*). The three type II and one type I calcium-binding sites are conserved in (**A** to **F**) and are labeled and color-coded according to their domains in (**C**) and (**G**).

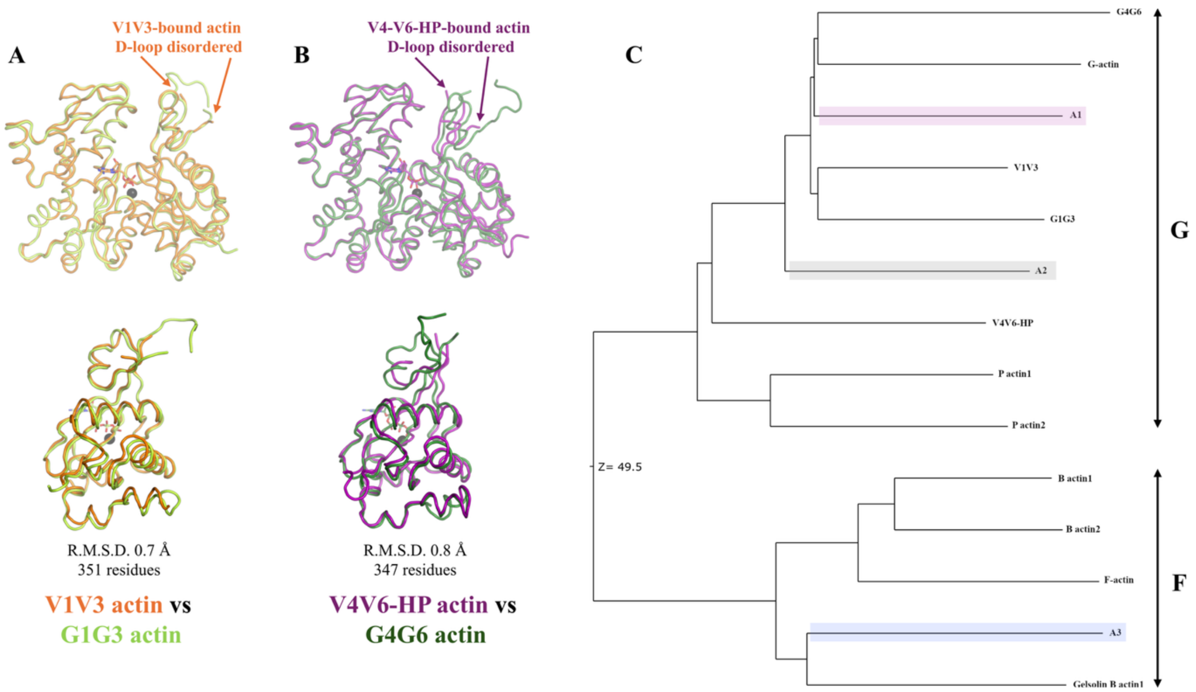

Fig. S13. Comparison of the villin- and gelsolin-bound actin conformations. Superimpositions of actin subunits from (**A**) the V1V3/actin complex (orange) with the G1G3/actin complex X-ray structure (light green, PDB ID 3FFK) (*45*) and (**B**) the V4V6-HP/actin complex (magenta) with the G4G6/actin complex X-ray structure (dark green, PDB ID 1H1V) (*23*). Actin adopts similar conformations when bound to the N-terminal or C-terminal halves of villin and gelsolin. (**C**) Dendrogram showing the structural relatedness of actin subunits (*68*). VA3 actin A3 groups with F-actin conformations. B actin1, B actin2 and F-actin refer to the sequential actin subunits from the cryo-EM structure of the actin filament barbed end (PDB ID 8F8R) (*25*). Gelsolin B actin 1 refers to the barbed-end subunit from the cryo-EM structure of the actin filament gelsolin-capped filament (*22*). VA3 A2 and A1 actins group with G-actin conformations. P actin1 and P actin2 refer to the sequential actin subunits from the cryo-EM structure of the actin filament pointed end (PDB ID 8F8S) (*25*). V1V3 and V4V6 refer to the actin subunits bound to these fragments reported here. G1G3 (PDB ID 3FFK) (*45*) and G4G6 (PDB ID 1H1V) (*23*) refer to the corresponding actin subunits from gelsolin/actin X-ray structures. G-actin is the native actin X-ray structure (PDB ID 3HBT) (*24*).

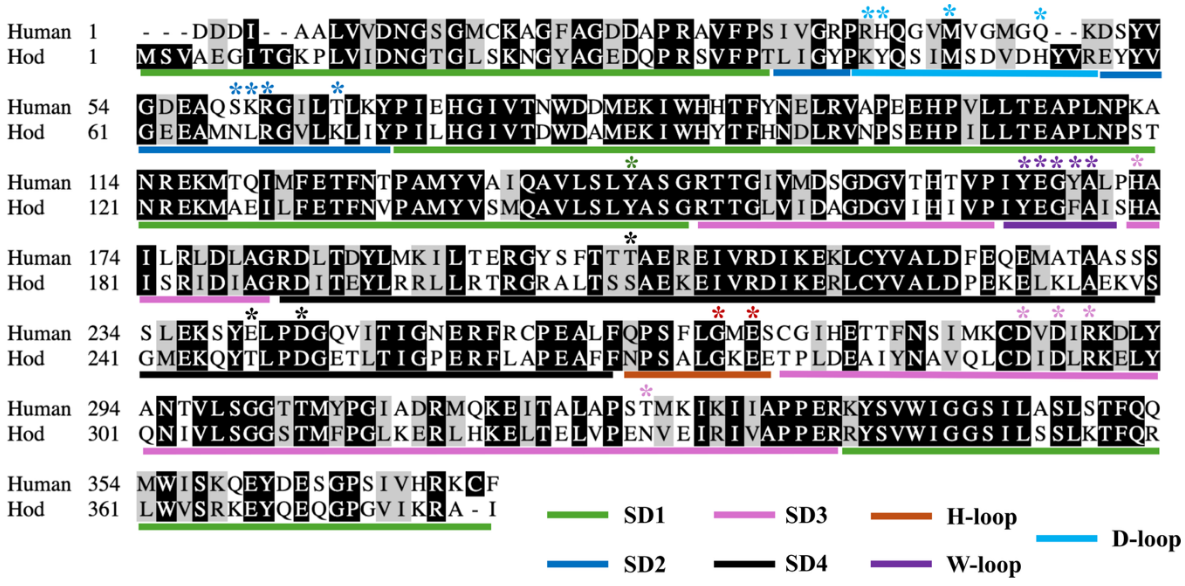

Fig. S14. Sequence alignment of human and Hodarchaeales actin. An example of a Hodarchaeales (Hod) actin (NHJ01685) aligned with human β-actin. Asterisks in shades of blue, light blue (D-loop) and dark blue (SD2), (2 conserved from 8 residues) and black (1 conserved from 3 residues) highlight residues from A3 SD2 and SD4, respectively, that are involved in the G-to-F flattening transition. Asterisks in brown (2 conserved from 2 residues), pink (4 conserved from 5 residues), purple (4 conserved from 5 residues), highlight the A2 H-loop, A1 SD3 residues, and the A1 SD3 W-loop, respectively, that induce the flattening transition. The asterisk in green indicates the conserved Tyr143 from the W-loop gate. Overall, comparing the G-to-F transition contact residues in these two sequences, 14 of 24 are absolutely conserved and 9 of 10 non-conserved residues are conservative substitutions, suggesting that the actin nucleus activation mechanism and W-loop gate evolved prior to eukaryogenesis.

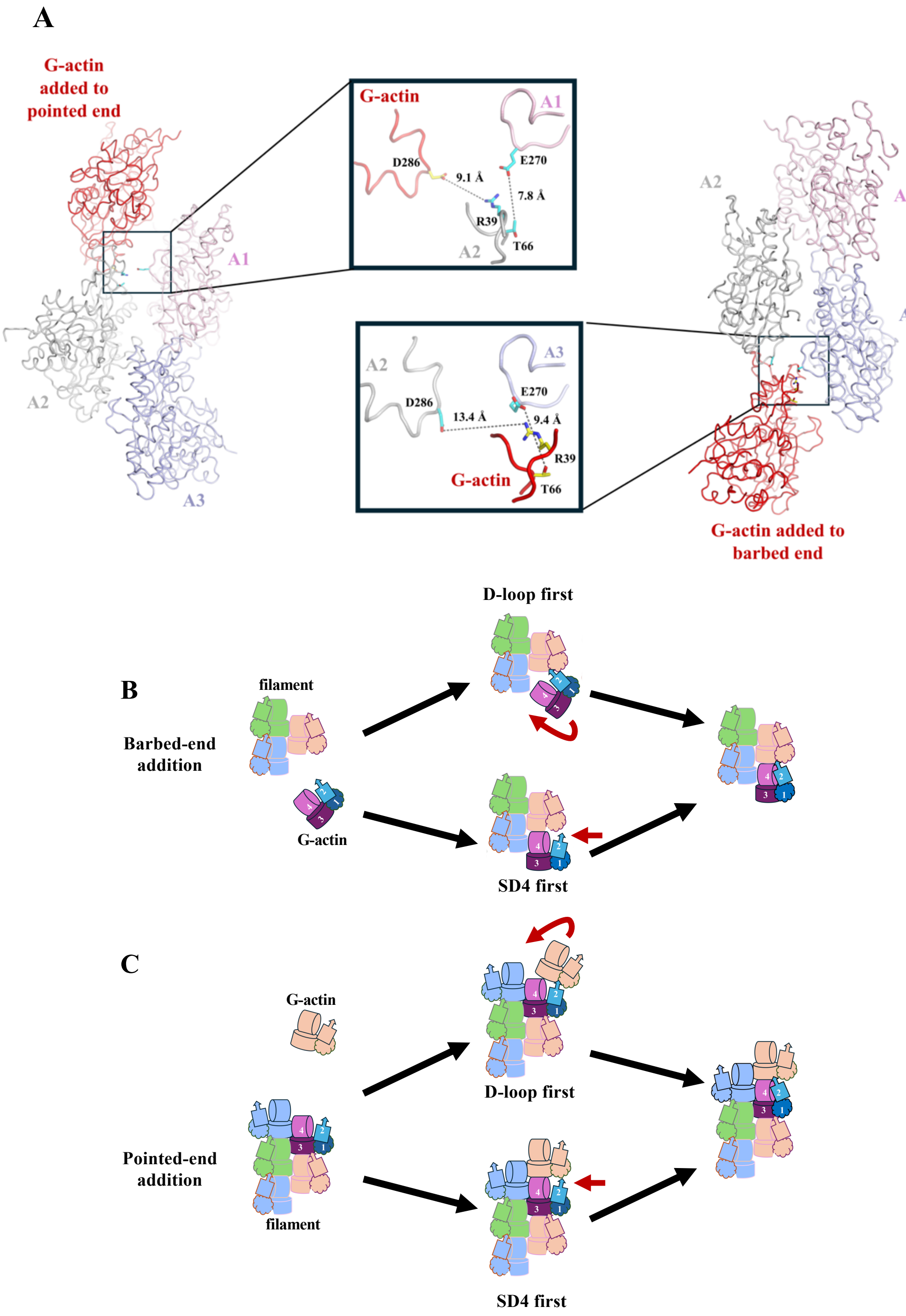

Fig. S15. Distances and docking modes. A) Distances between critical residues in the models from Fig. 7. Respective distances from the H-loop Glu270 and SD3 Asp286 to the Thr66 and Arg39 of the actin subunit undergoing the G-to-F transitions are similar, but slightly shorter, in the pointed-end addition compared to the barbed-end addition (see enlargements). (**B** and **C**) Cartoon representations of potential docking modes at the barbed and pointed ends, respectively. Red arrows indicate the flattening transition. The flattening subunit is colored in shades of blue and purple with the subunits labeled. This 2D cartoon is intended to show the differences in the hypothetical docking modes within a single strand. The correct interstrand connections are lost in making the 2D cartoon from the 3D filament and should not be interpreted.

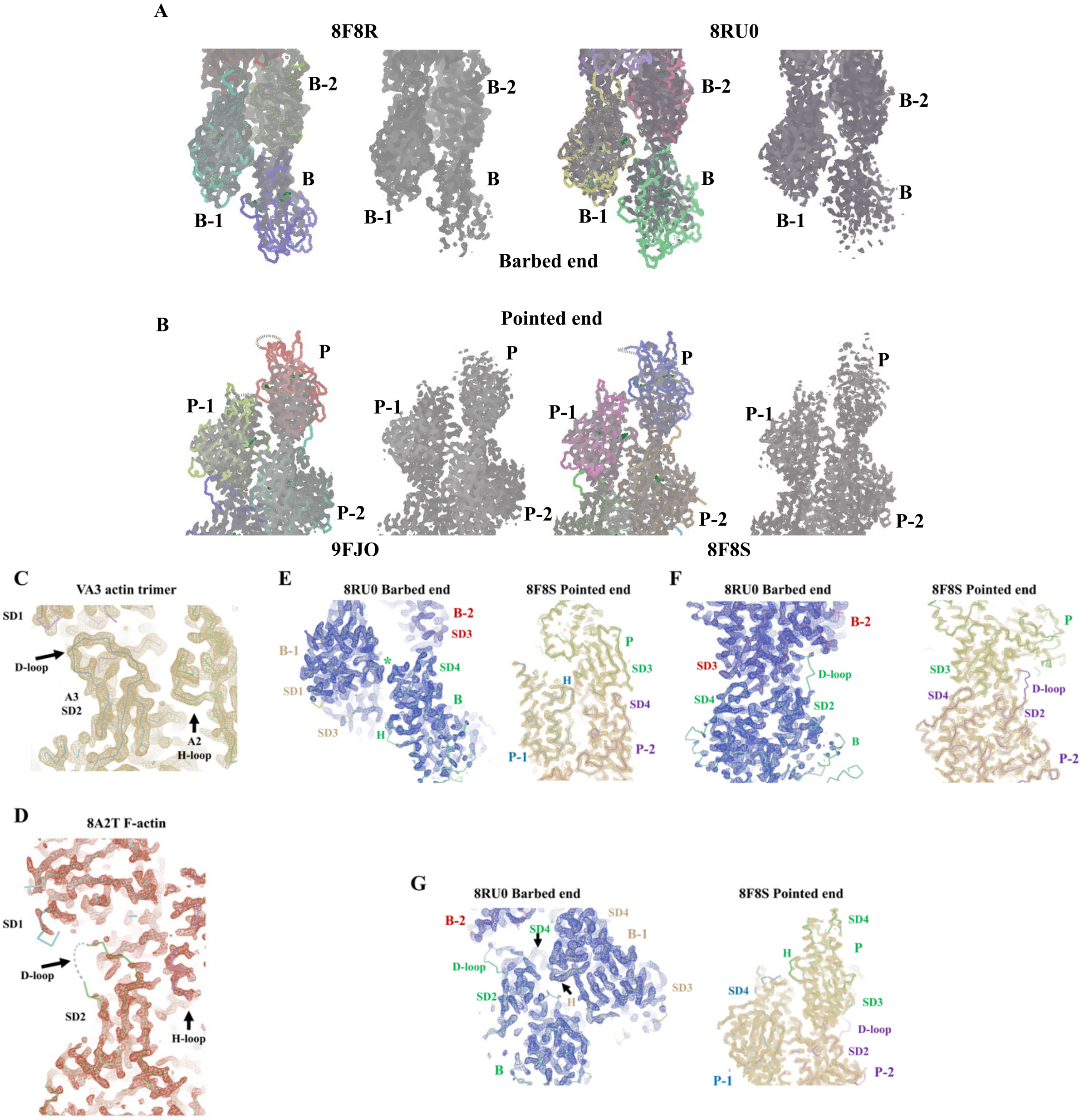

Fig. S16. Density maps. Dynamic regions of F-actin indicated from cryo-EM maps. (**A**) Barbed end. PDB IDs 8F8R (at 3.3 Å resolution, (*25*)) and 8RU0 (at 3.08 Å resolution, (*1*)) are contoured at RMSD 11.3 and 13.7, respectively. (**B**) Pointed end. PDB IDs 9FJO (at 3.3 Å resolution, (*31*)) and 8F8S (at 2.84 resolution, (*25*)) are contoured at RMSD 11.3 and 13.7, respectively. The density maps (gray) in **A** and **B** are shown in duplicate, with and without the models. These maps indicate mobility in the terminal subunit, due to the relatively low levels of density with respect to the subsequent subunits. (**C**) The A3 D-loop in the VA3 X-ray composite OMIT map contoured at 2 σ. The D-loop shows comparable electron density to the rest of SD2. (**D**) The D-loop in a cryo-EM map. PDB ID 8A2T (at 2.24 resolution, (*1*)) contoured at RMSD 10.0. At this contour level there is no density for the D-loop whilst the remainder of SD2 is has well defined density, indicating that the D-loop site is partially disordered due to dynamics. (**E**-**G**) Density maps from the cryo-EM barbed (8RU0) and pointed (8F8S) ends contoured at RMSD 11.0 and 9.5, respectively, to highlight the relative stability of interfaces: SD3:SD4 and SD3:SD2 longitudinal contacts are characterized by strong density, whereas the D-loops have little or no density; lateral H-loop contacts (H) and lateral helix contacts (asterisk) show stronger density that D-loops at this contour level, indicating greater stability.

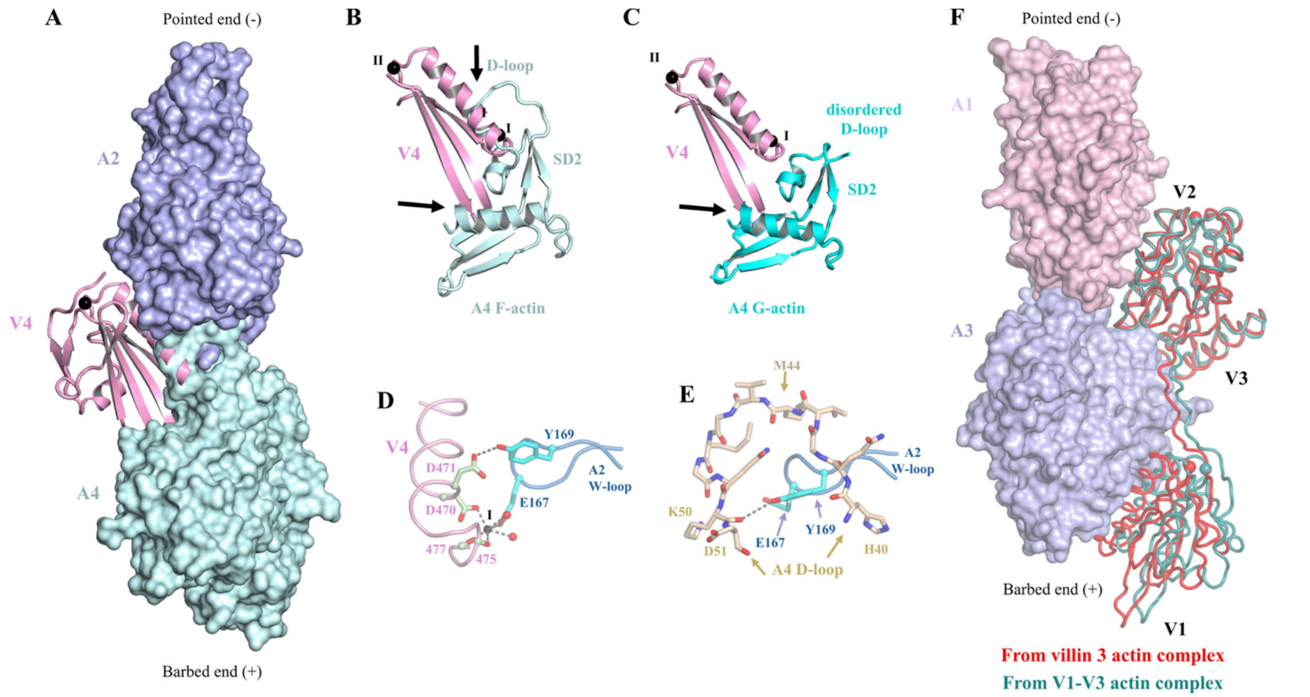

Fig. S17. Contribution of V4 to actin filament severing. V4 has similar steric clashes to V1 (Fig. 8, A to E) when superimposed onto F-actin (*25*). (**A**) Superimposition of V4 onto two longitudinally related subunits of F-actin (A2 and A4). Major steric clashes are seen between the V1 long helix and A4 SD2. (**B**) A cartoon representation of the clashing region showing that the steric hindrance with the D-loop is lost when F-actin A4 is replaced by G-actin (**C**). Black arrows indicate steric clashes. (**D**) In the villin:actin complex, A2 SD3 W-loop residues Glu167 and Tyr169 respectively coordinate the type I calcium and Asp471 from villin. (**E**) In F-actin, A2 SD3 W-loop residue Tyr169 coordinates the A4 carbonyl oxygen from D-loop residue 50. (**F**) Superimposition of V1V3 from the V1V3/actin structure, based on overlaying domains V2-V3, onto the VA3 structure. V4V6-HP and actin A2 are omitted for clarity. The V1 domains display different relative positions, implying flexibility in the V1V2 linker.

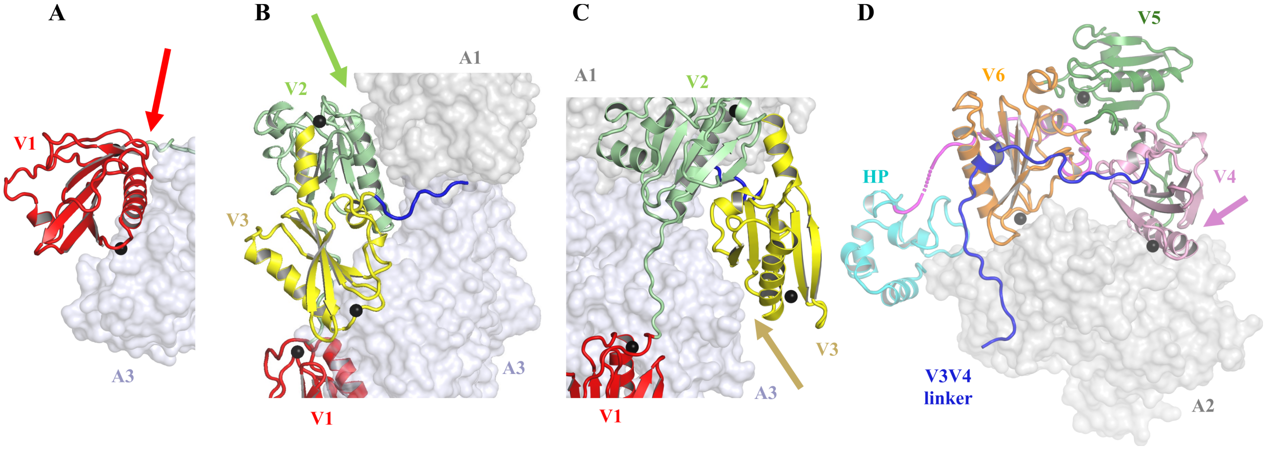

Fig. S18. Close packing of villin domains with actin. (**A**) V1 interacts tightly with actin A3 through its long helix (red arrow). (**B**) V2 forms a relatively distant interaction with A1 (green arrow). (**C**) V3 forms a relatively distant interaction with A3 (yellow arrow). (**D**) V4 interacts tightly with A2 through its long helix (pink arrow). V5 does not interact with actin, while V6 forms a minor interaction with A2. The villin HP domain forms relatively minor interactions with A2 and A3 (Fig. 1E and fig. S7, A and B). The buried areas on the actin protomers are A3 1950 Å^2, A2 1722 Å^2 and A1 954 Å^2.

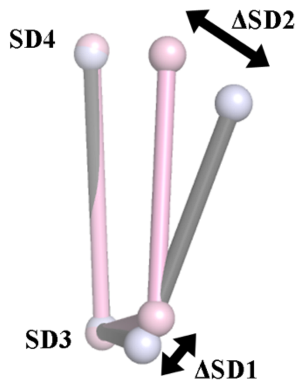

Fig. S19. Definition of parameters in Table 1. ϕ SD1-4: The dihedral angle between the center of masses (COM) that link SD3-SD4 and SD1-SD2. **Δ SD1:** refers to the distance between the COM of SD1 to the COM of SD1 from G-actin or F-actin, after SD3-SD4 have been superimposed. The angle is that between SD1-SD3 and SD1 from G-actin or F-actin. **Δ SD2:** refers to the distance between the COM of SD2 to the COM of SD2 from G-actin or F-actin, after SD3-SD4 have been superimposed.

Table S1. X-ray data collection and refinement statistics.

	VA3 (PDB ID 9JUS)	V1V3/Actin (PDB ID 9JW0)	V4V6-HP/Actin (PDB ID 9JVT)
**Crystals**			
Crystallization conditions	0.1 M Bis-Tris propane, pH 6.5<br>10% PEG 3350<br>0.2 M KSCN<br>5 mM $CaCl_2$/$MgCl_2$	0.1 M Tris, pH 8.0<br>15% PEG 3350<br>1 mM $CaCl_2$	0.1 M Tris, pH 8.0<br>50% PEG 400<br>0.2 M Li_2SO_4<br>1 mM $CaCl_2$
Lattice	P1	$P22_12_1$	C2
a, *b*, *c* (Å)	98.1, 102.4, 147.1	84.7, 93.8, 114.3	131.1, 97.7, 134.2
α, β, γ (°)	77.9, 72.6, 65.0	90, 90, 90	90, 104.0, 90
**Data collection**			
Beamline	BL41XU, SPring-8	TPS 05A, NSRRC	BL41XU, SPring-8
Wavelength (Å)	1.0	1.0	1.0
Resolution (Å)	29.9-2.7 (2.75-2.70)	20.0-2.69 (2.75-2.69)	46.6-3.29 (3.52-3.29)
R_{merge}	8.1 (123.2)	15.6 (128.3)	12.4 (99.1)
R_{meas}	9.6 (145.0)	16.8 (141.1)	14.6 (116.3)
R_{pim}	5.1 (75.6)	6.3 (57.6)	7.7 (60.5)
$I/\sigma(I)$	7.2 (1.0)	13.2 (1.1)	7.6 (1.3)
$CC_{1/2}$	(0.534)	(0.459)	(0.570)
Completeness (%)	98.3 (97.9)	100.0 (99.4)	99.5 (97.9)
Redundancy	3.5 (3.6)	7.1 (5.8)	3.5 (3.6)
**Refinement**			
Resolution (Å)	29.9-2.70 (2.80-2.70)	19.9-2.69 (2.79-2.69)	45.6-3.29 (3.41-3.29)
No. reflections	133624 (13218)	24117 (1423)	24931 (2404)
R_{work} / R_{free}	19.6/22.6 (34.4/38.1)	20.3/25.9 (26.8/30.30)	24.1/26.8 (38.1/42.5)
No. atoms			
Protein	30201	5603	12102
Ligands	188	36	64
Water	187	92	0
B factors			
Protein	107.2	53.0	125.3
Ligands	80.1	37.8	93.2
Water	77.2	40.4	0
r.m.s deviations			
Bond lengths (Å)	0.002	0.002	0.003
Bond angles (°)	0.50	0.49	0.60
Ramachandran Plot			
Favored (%)	96.71	96.71	94.41
Outliers (%)	0.16	0.29	0.40

Values in parentheses are for the highest-resolution shell

Movie Legends

Movie S1. The VA3 complex and superimposition onto F-actin. VA3 is depicted as in Fig. 1A. F-actin is shown in cartoon format (blue). The first section of the movie is an animated version of Fig.1 A-D, showing the geometry of the VA3 complex. The final section (at 14 s) of the movie shows an animated version of Fig. 3B, highlighting that the orientation of the actin subunits within that VA3 are similar to the geometry of the filament. Villin domains V1 and V4 block addition of actin monomers to the filament at the barbed end, while actin subunits A1 and A2 are available for pointed-end elongation.

Movie S2. Comparison of activated villin and gelsolin structures. The actin-nucleus bound villin is depicted as in Fig. 1A, and gelsolin from the cryo-EM structure of the gelsolin-capped actin filament (PDB ID 8VIZ) is colored gray (*22*). Actin subunits are omitted for clarity. Calcium ions are shown as black spheres. Villin and gelsolin domains show similar relative positions with some differences observed in linker regions.

Movie S3. Comparisons of actin subunit conformations. The movie shows a morph between various actin subunit conformations: from G-actin (dark green, PDB ID 3HBT, X-ray structure) to V4V6-HP bound actin (light green, at 4 s); to A2 (gray, at 7 s); to the pointed-end P-1 actin from the cryo-EM structure (mauve, PDB ID 8F8S, at 10 s), back to G-actin (dark green, PDB ID 3HBT, X-ray structure at 13 s); to V1V3 bound actin (gold, at 16 s); to A1 (pale pink, at 19 s); to the pointed-end P actin from the cryo-EM structure (sky blue, PDB ID 8F8S, at 22 s), back to G-actin (dark green, PDB ID 3HBT, at 25 s); to A3 (slate blue, at 28 s); to the cryo-EM structure of F-actin (cyan, PDB ID 8A2T, at 31 s); back to A3 (slate blue, at 34 s); to the barbed-end actin from the cryo-EM structure of the gelsolin-capped filament (pink, PDB ID 8VIZ, at 37 s); back to A3 (slate blue, at 40 s); to the barbed-end actin from the cryo-EM structure of the filament (lime, PDB ID 8RU0, at 43 s); back to A3 (slate blue, at 46 s). In general, SD3 and SD4 are largely static in the movie, while SD1 and SD2 adopt different relative positions in the "G-actin" twisted structures, and show larger relative shifts in moving to "F-actin" flattened conformations, including ordering of the D-loop. A3 adopts a conformation between F-actin and the gelsolin-bound conformation. Residues in the W-loop gate are depicted, highlighting the open and closed conformations. The SD1 and SD4 helices (which form a lateral contact in F-actin) are labeled, with the SD1 helix exhibiting conformational displacement during the various transitions.

Movie S4. 600 ns MD simulation of the ATP-bound VA3 actin trimer. The MD frames are superimposed on to the three terminal subunits of the structure of the pointed end of the filament (gray) using trimer subunit A3 (*25*). A3 (brown, left bottom), A2 (peach, right), A1 (pink, left top). The 18 s movie covers the entire 600 ns simulation. During the first 400 ns (0-12 s of the movie) the trimer samples conformations, some of which are similar to the pointed-end template (gray). After 400 ns A2 begins to dissociate leaving the dimer (A1/A3) intact.

Movies S5A,B. Determinants of the G-to-F transition in the actin nucleus. The movies show morphs between the hypothetical docking of A1 and A3 during dimer formation. Initially A1 and A3 are shown in the G-actin conformation, which morph into the experimentally determined A1 and A3 conformations on dimer coalescence (step 1 Fig. 6A), followed by trimer formation with A2 (H-loop and SD1 helix are shown only), where it is stabilized by the interactions shown in Fig. 5, B and C (step 2 Fig. 6A). Because A2 is not in an F-actin conformation, its SD1 helix cannot engage the A3 SD4 helix to form a lateral interaction. The A1 W-loop gate (labeled) undergoes rearrangement as Tyr143 disengages from the mainchain carbonyl of residue 168, which in turn binds to Gln49, thereby opening the Met44 binding pocket. (**A**) The "SD4-first" scenario, initial SD4-SD3 contact is followed by A1-induced conformational changes in A3, enabling the A3 D-loop to become ordered by inserting Met44 into its binding site on A1. (**B**) The "D-loop-first" scenario, insertion of the A3 D-loop precedes a swing of A3, allowing SD4 of A3 to contact SD3 of A1. The docking of the D-loop and

the incoming actin is taken from the cryo-EM structure of the incoming profilin:actin at the barbed end of INF2 bound filament (PDB ID 9AZP) (*35*). This template for "D-loop" first docking is used in subsequent movies.

Movie S6. Model of nucleation and initial elongation and depolymerization. The movie shows an animated version of Fig. 6, A and B. The movie shows the formation of the dimer and trimer and follows the fate of the actin trimer nucleus during polymerization/depolymerization. The conformational changes are modelled by morphing between G-actin (*24*) and A3 or F-actin structures (*25*). Conformational dynamics in the monomer conformations and at filament ends are modelled to highlight the potential roles in actin dynamics. The conformational dynamics are exaggerated to be obvious in the movie. Fluctuations will be present in all subunits but are not shown. Rotating animations are provided to give different views of the polymerization/depolymerization. **Step 1, Dimer formation (7-13 s):** Two conformationally fluctuating monomers come together via A3 SD4 interacting with A1 SD3. A3 is likely to have partially fluctuated along the flattening transition at the point of contact, after which template-induced (A1) transition will complete the G-to-F transition of A3 (movie S5). Docking of the A3 D-loop also requires conformational fluctuation in A1 to open the W-loop gate and reveal the A1 Met44 binding site. A1 remains in a twisted conformation. **Step 2, Trimer formation (13-15 s):** A2 approaches laterally, and its H-loop stabilizes the flattened conformation of A3 (movie S5). A1 and A2 remain in twisted conformations. **Step 3, Barbed end addition (15-17 s):** G-actin SD4 docks onto A2 (via SD3) and A3 (via two H-loop interactions and one lateral helix zone interaction) (movie S7). A2 and A3 provide the template to induce flattening of the docked monomer (**Step 4, 17-20 s**). Conformational fluctuation in A2 is required to open its W-loop gate and allow D-loop binding. A1 and A2 remain in twisted conformations and now represent the P and P-1 subunits at the pointed end of a filament. A3 and the fourth actin are in flattened conformations and represent B-1 and B subunits at barbed end of a filament. **Steps 5 and 6, Barbed end addition and G-to-F transition (20-24 s):** G-actin docks (movie S10) and transitions to the flattened form as in steps 3 and 4 (movie S8). At this stage the A3 W-loop gate should be open, as observed in the cryo-EM structure of the barbed end (PDB ID 8RU0). Subsequent barbed end additions will proceed via repetition of steps 5 and 6 (movie S10). **Step 7, Pointed end addition and G-to-F transition (24-28 s):** G-actin SD3 docks onto A2 (via SD4) and A1 (via two H-loop interactions, no lateral helix zone interaction, movie S11). A2 undergoes template-induced flattening (movie S9), with the template consisting of three subunits (A3, A1 and the docked monomer). The A3 provides a lateral helix contact, A1 provides the H-loop, and the docked monomer provides the D-loop binding site to stabilize A2 in a flattened form. Conformational fluctuation in the docked monomer is required to open its W-loop gate and allow A2 D-loop binding. We suggest that the lack of a lateral helix contact for G-actin docking and conformational change, relative to the barbed end, is the major reason for slower elongation at the pointed end. **Steps 8 and 9, Pointed end addition and G-to-F transition (28-32 s):** Subsequent pointed end additions will proceed via repetition of step 7. **Pointed end dissociation (32-37 s):** P subunit fluctuations weaken lateral H-loop interactions with P-1. However, the twisting due to the F-to-G conformational change occurs in the P-2 subunit. P-2 is surrounded by four other subunits. The P-3 lateral helix contact, the P-1 H-loop, and the D-loop binding site on dissociating subunit all stabilize P-2 in a flattened form. Lateral fluctuations in the P-2 subunit relative to P-1 and P-3 (not shown) likely occur to relieve P-2 of the lateral stabilizing contacts allowing the F-to-G transition twist of P-2 SD2/D-loop relative to SD4 to disrupting the P-actin binding site leading to its dissociation. **Barbed end dissociation (38-45 s):** The B subunit flattened form is stabilized by lateral interactions to B-1. Fluctuations will weaken lateral contacts, particularly by dissociating the B-1 H-loop to B SD2 interaction which if present stabilizes the flattened conformation, to allowing the F-to-G transition twist of B SD2/D-loop relative to SD4 to disrupting its interaction with B-2 leading to B-subunit dissociation. **Final trimer dissociation (47-50 s):** The lateral subunit (A2) dissociates before the longitudinal separation of A1/A3.

Movie S7. Docking G-actin onto the actin nucleus. An animated version of Fig. 7A and B, highlighting the lateral helix contacts. The possible two extremes of initial docking contacts are depicted, "SD4 first" and "D-loop first". No steric clashes are observed in these docking orientations. At the barbed end (9-11 s), "SD4 first" initial docking forms one longitudinal contact (G-actin SD4 to A2 SD3), one full lateral helix contact (G-actin SD4 to A3 SD1 at *), and two partial H-loop contacts G-actin H-loop to A3 (with His173 not shown), and G-actin (Thr203 not shown) to A3 H-loop (Glu270 not shown). Whereas "D-loop first" initial docking forms one longitudinal contact (G-actin SD2 and D-loop to A2 SD3) and one full H-loop contact (G-actin H-loop to A3 SD2 and SD4). Both initial docking orientations have merits: "SD4 first" can dock as a rigid body onto a relatively wide docking site, whereas "D-loop first" requires an open W-loop gate and ordering of the D-loop, but achieves a more compact binding site with greater buried surface area. We suggest both orientations may be possible. At the pointed end (18-20 s), the models only predict longitudinal interactions: "SD4 first" G-actin SD3 to A2 SD4; and "D-loop first" G-actin SD3 to A2 SD2 and D-loop. Both initial docking orientations require conformational flexibility to narrow the distance between A1 and G-actin in order to form lateral interactions, to favor pointed end elongation over nucleation dimer formation, where no lateral interactions are possible.

Movies S8A,B. Determinants of the G-to-F transition at the barbed end. The movies show morphs between the hypothetical docking of G-actin onto the barbed-end subunits B and B-1, as described in Fig. 7, during barbed-end elongation followed by the G-to-F conformational change (steps 5-6 Fig. 6A). The morph transitions G-actin (X-ray structure, PDB ID 3HBT) (*24*) and B and B-1 barbed-end subunits to B, B-1 and B-2 barbed-end subunits, respectively, from barbed end cryo-EM structure which has an open W-loop gate (PDB ID 8RU0) (*28*). Only the H-loop (left view) or H-loop and SD1 helix (right view) are shown from initial B subunit for clarity. (**A**) The "SD4 first" scenario, initial SD4-SD3 contact is followed by B-1 induced conformational changes in G-actin, enabling the G-actin D-loop to become ordered by inserting Met44 into its binding site on B-1. (**B**) The "D-loop first" scenario, the D-loop docked G-actin is taken from PDB ID 9AZP (*35*). Insertion of the G-actin D-loop precedes a swing of G-actin, allowing SD4 of the flattening G-actin to contact SD3 of B-1 and SD1 of the B subunit. Note addition of the fourth subunit to the nucleus encounters an opening and closing W-loop gate (similar to movies S5A,B), whilst later additions encounter an open gate. These movies differ from movies S5A,B in that the B and B-1 are in F-actin-like conformations and are in place before G-actin docks, allowing the B subunit SD1 helix to provide an extra component to the G-actin docking site.

Movies S9A,B. Determinants of the G-to-F transition at the pointed end. The movies show morphs between the hypothetical docking of G-actin onto the pointed-end subunits P and P-1, as described in Fig. 7, during pointed-end elongation, followed by the G-to-F conformational change (steps 1-2 Fig. 6B). The morph transitions G-actin (X-ray structure, PDB ID 3HBT (*24*)) and P and P-1 barbed-end subunits to P, P-1 and P-2 barbed-end subunits, respectively, from pointed end cryo-EM structure (PDB ID 8F8S (*25*)). Only the H-loop (left view) or H-loop and SD1 helix (right view) are shown from initial P subunit for clarity. (**A**) The "SD4 first" scenario, initial SD4-SD3 contact is followed by G-actin induced conformational changes in P-1, enabling the P-1 D-loop to become ordered by inserting Met44 into its binding site on the incoming G-actin. (**B**) The "D-loop first" scenario, A1 is docked on to the P-1 actin D-loop using PDB ID 9AZP (*35*) as a template. Insertion of the P-1 D-loop, into the incoming G-actin Met44 binding pocket, precedes a swing of G-actin, allowing SD4 of the flattening P-1 to contact SD3 of P-2 and SD1 of the P subunit. These movies differ from movies S5A,B and S8A,B in that the P and P-1 subunits are in G-actin-like conformations and are in place before G-actin docks. The P subunit SD1 helix is too distant to provide a G-actin docking site, since the P subunit has not undergone the G-to-F transition. The G-actin subunit needs to dynamically open the W-loop gate to accept Met44 from the P-1 actin.

Movie S10. Docking of G-actin at the barbed end. An animated version of the model in Fig. 7B, highlighting the lateral helix contacts (see Fig. 7C), but shown for both hypothetical docking scenarios. In "SD4 first" docking, G-actin SD4 (cyan, PDB ID 3HBT (*24*)) is docked onto B-1 SD3 (dark purple) of the barbed end of actin filament (PDB ID 8RU0 (*28*)) by superimposition on the F-actin structure (PDB ID 8A2T (*1*)), no steric clashes are present. The movie proceeds to highlight the three barbed-end lateral contact zones to the docked G-actin: the G-actin H-loop (H1) with B-subunit His173 (see movie S8A); the contact between G-actin SD4 helix and B-subunit (mauve) SD1 helix (asterisk); and the B-subunit H-loop (H2) Glu270 contact to G-actin residue Thr203 (see movie S8A). The B-subunit H-loop is positioned to stabilize the G-actin SD2 G-to-F transition (movie S8A). The B actin subunit is additionally stabilized by a further two lateral contact zones to B-1 actin: B-subunit SD4 helix to subunit B-1 SD1 helix, and the B-1 H-loop (H3) stabilizing the F-actin conformation of the B-subunit SD2 and D-loop. Furthermore, the H-loop (H2) contact zone expands to include His173 (not shown) from subunit B-1. Together these interactions form five lateral contact zones, with the B-subunit H2 to G-actin contact initially partial and completed upon G-to-F transition. Subsequent subunits (B-1, B-2 and B-3) engage the full set of five lateral contact zones.

In "H-loop first" docking, the G-actin D-loop (light blue, PDB ID 9AZP (*35*)) is docked onto B-1 SD3 (dark purple) of the barbed end of actin filament (PDB ID 8RU0 (*28*)) by superimposition on the F-actin structure (PDB ID 8A2T (*1*)), no steric clashes are present. Residue details are found in movie S8B. In this model after the initial docking the added subunit swings and flattens to make the lateral contacts described in the "SD4 first" model.

Movie S11. Docking of G-actin at the pointed end. An animated version of the model in Fig. 7A, highlighting lateral helix contacts (see Fig. 7C). In "SD4 first" docking, G-actin SD3 (orange, PDB ID 3HBT (*24*)) is docked onto the P-1 SD4 (gray) of the pointed end of the actin filament (PDB ID 8F8S (*25*)) by superimposition on to the F-actin structure (PDB ID 8A2T (*1*)), no steric clashes are present. The movie proceeds to highlight the loss of lateral contacts in moving from P-3 to the terminal docked G-actin. Residues are not shown in the movie but can be found in movie S9A. P-3 has the full complement of five lateral contact zones, including its H-loop (H1) with P-2 His173, the P-3 SD4 helix with the P-2 SD1 helix; and stabilization of its SD2 and D-loop by the P-2 H-loop (H2). The contacts to P-4 are not shown. P-2 lacks the SD4 helix to P-1 SD1 helix contact because P-1 does not adopt an F-actin conformation. Thus, the four P-2 lateral contacts are: P-2 His173 with the P-3 H-loop (H1); P-2 SD1 helix with P-3 SD4 helix; P-2 H-loop (H2) with the P-3 subunit SD2/D-loop and with P-1 His173; and P-2 subunit SD2 and D-loop with P-1 H-loop (H3). P-1 is missing the SD4 helix to P SD1 helix contact, since the P subunit does not adopt an F-actin conformation, leaving three lateral contacts: P-1 His173 with P-2 H-loop (H2), P-1 H-loop (H3) with P-2 SD2/D-loop and P His173; and P-1 SD2 residue Thr203 with P H-loop (H4) Glu270. The latter is only partial, as P-1 is not in an F-actin conformation and its D-loop does not contact the P H-loop. The P subunit also lacks the P SD4 helix to G-actin SD1 helix contact. Thus, the three P lateral contacts are: P His173 with P-1 H-loop (H3), P H-loop (H4) with G-actin His173, and the P H-loop Glu270 with P-1 Thr203, again a partial interaction. In this static model, the G-actin H-loop (H5) Glu270 distance to P actin Thr203 is too distant for bonding. Other interactions from P-1 subunit residue Lys191 (NZ) to P subunit residue Ile175 (O), and from Ser199 (OG) to Asp179 (OD2) or to Arg177 (NH2) may also stabilize the two terminal subunits (*25*). Finally, the docked G-actin forms a single lateral contact, His173 with the P H-loop (H4, and movie S9A).

In "H-loop first" docking, A1 is docked on to the P-1 actin D-loop via superimposition onto the pointed end of the actin filament (PDB ID 8F8S (*25*)) using PDB ID 9AZP (*35*) as a template, no steric clashes are present. Residue details are found in movie S9B. In this model, after the initial docking the added subunit swings while subunit P-1 flattens to make the lateral contacts described in the "SD4 first" model.

Movie S12. Clashes between V1 and F-actin. The movie shows a rotation of Fig. 8A, highlighting the steric clash between V1 and F-actin in the superimposed model.

Movie S13. The reduced clashes between V1 and G-actin relative to F-actin. The movie shows an animated version of the proposed mechanism of the F-to-G transition during actin-filament severing by villin (Fig. 8, B and C).

Movie S14. Model of villin severing. The movie shows an animated version of the proposed mechanism of actin-filament severing by villin (Fig. 9). The F-to-G transition at the severing position weakens both longitudinal and lateral contacts destabilizing the filament connection (movie S15).

Movie S15. Consequences of a G-actin conformation within a filament. G-actin (green, PDB ID 3HBT (*24*)) is docked onto an F-actin subunit P-1 subunit within a filament (pink, PDB ID 8A2T (*1*)), and the movie shows a morph between the structure and model. This hypothetical model has two implications. Firstly, the dissociation of the D-loop weakens longitudinal contacts. Secondly, the loss of a lateral helix contact and part of the H-loop lateral contact with the D-loop weakens lateral contacts.

Movie S16. Model of A3 in the filament. The movie shows a morph between an F-actin subunit (pink), in a filament (cyan, (*25*)), to the A3 conformation (slate blue). A3 was overlaid on the F-actin subunit using SD3 and SD4. This modelled conformational change produces potential alterations in actin subunit interactions, in the D-loop interactions from the lower actin and in the cross-filament interactions, due to the movement of SD1 (black asterisk) from A3 relative to F-actin. The position of actin SD1 in A3, in the VA3 structure, is influenced by V1 and V3 binding. However, this structure cannot be used to distinguish the relative contributions during severing, since V1 has adopted the capping position in the VA3 structure, which is equivalent of the endpoint structure after severing has occurred. If V3 induces part of the conformational change in SD1 during the phase of villin attachment to the filament (Fig. 9), as hypothesized from the cryo-EM structure of the gelsolin-capped filament (*22*), then we suggest that this will aid D-loop dissociation and cross-filament propagation of the actin conformational changes during villin severing, by disengaging the helix zone lateral contact. Asterisks indicate the helix zone cross filament contacts in the movie (Fig. 3A and fig. S6).

At the end of the movie the five lateral zone interactions of a single subunit within F-actin are labeled: 3 H-loop zones (H1-H3) and 2 helix zones (red and black asterisks). Contacts between two laterally-related subunits are limited to three interaction zones: 2 H-loop zones (H1-H2) and 1 helix zone (black asterisk); or similarly, due to symmetry, H-loop zones H2 and H3, and 1 helix zone (red asterisk).

REFERENCES AND NOTES

1. W. Oosterheert, B. U. Klink, A. Belyy, S. Pospich, S. Raunser, Structural basis of actin filament assembly and aging. *Nature* **611**, 374–379 (2022).

2. L. Blanchoin, R. Boujemaa-Paterski, C. Sykes, J. Plastino, Actin dynamics, architecture, and mechanics in cell motility. *Physiol. Rev.* **94**, 235–263 (2014).

3. A. Wegner, J. Engel, Kinetics of the cooperative association of actin to actin filament. *Biophys. Chem.* **3**, 215–225 (1975).

4. A. D. Rosenbloom, E. W. Kovar, D. R. Kovar, L. M. Loew, T. D. Pollard, Mechanism of actin filament nucleation. *Biophys. J.* **120**, 4399–4417 (2021).

5. D. Sept, J. A. McCammon, Thermodynamics and kinetics of actin filament nucleation. *Biophys. J.* **81**, 667–674 (2001).

6. T. D. Pollard, Rate constants for the reactions of ATP- and ADP-actin with the ends of actin filaments. *J. Cell Biol.* **103**, 2747–2754 (1986).

7. T. Kotila, H. Wioland, G. Enkavi, K. Kogan, I. Vattulainen, A. Jegou, G. Romet-Lemonne, P. Lappalainen, Mechanism of synergistic actin filament pointed end depolymerization by cyclase-associated protein and cofilin. *Nat. Commun.* **10**, 5320 (2019).

8. N. Courtemanche, Mechanisms of formin-mediated actin assembly and dynamics. *Biophys. Rev.* **10**, 1553–1569 (2018).

9. P. W. Gunning, U. Ghoshdastider, S. Whitaker, D. Popp, R. C. Robinson, The evolution of compositionally and functionally distinct actin filaments. *J. Cell Sci.* **128**, 2009–2019 (2015).

10. T. D. Pollard, Actin and actin-binding proteins. *Cold Spring Harb. Perspect. Biol.* **8**, a018226 (2016).

11. K. Zaremba-Niedzwiedzka, E. F. Caceres, J. H. Saw, D. Backstrom, L. Juzokaite, E. Vancaester, K. W. Seitz, K. Anantharaman, P. Starnawski, K. U. Kjeldsen, M. B. Stott, T.

Nunoura, J. F. Banfield, A. Schramm, B. J. Baker, A. Spang, T. J. Ettema, Asgard archaea illuminate the origin of eukaryotic cellular complexity. *Nature* **541**, 353–358 (2017).

12. U. Ghoshdastider, D. Popp, L. D. Burtnick, R. C. Robinson, The expanding superfamily of gelsolin homology domain proteins. *Cytoskeleton* **70**, 775–795 (2013).

13. C. Akıl, L. T. Tran, M. Orhant-Prioux, Y. Baskaran, E. Manser, L. Blanchoin, R. C. Robinson, Insights into the evolution of regulated actin dynamics via characterization of primitive gelsolin/cofilin proteins from Asgard archaea. *Proc. Natl. Acad. Sci. U.S.A.*, **117**, 19904–19913 (2020).

14. S. Nag, M. Larsson, R. C. Robinson, L. D. Burtnick, Gelsolin: The tail of a molecular gymnast. *Cytoskeleton* **70**, 360–384 (2013).

15. E. Friederich, K. Vancompernolle, D. Louvard, J. Vandekerckhove, Villin function in the organization of the actin cytoskeleton. Correlation of in vivo effects to its biochemical activities in vitro. *J. Biol. Chem.* **274**, 26751–26760 (1999).

16. A. Weber, J. Northrop, M. F. Bishop, F. A. Ferrone, M. S. Mooseker, Nucleation of actin polymerization by villin and elongation at subcritical monomer concentration. *Biochemistry* **26**, 2528–2536 (1987).

17. E. Yokota, M. Tominaga, I. Mabuchi, Y. Tsuji, C. J. Staiger, K. Oiwa, T. Shimmen, Plant villin, Lily P-135-ABP, possesses G-actin binding activity and accelerates the polymerization and depolymerization of actin in a Ca^{2+}-sensitive manner. *Plant Cell Physiol.* **46**, 1690–1703 (2005).

18. E. Ferrary, M. Cohen-Tannoudji, G. Pehau-Arnaudet, A. Lapillonne, R. Athman, T. Ruiz, L. Boulouha, F. El Marjou, A. Doye, J.-J. Fontaine, C. Antony, C. Babinet, D. Louvard, F. Jaisser, S. Robine, In vivo, villin is required for Ca^{2+}-dependent F-actin disruption in intestinal brush borders. *J. Cell Biol.* **146**, 819–830 (1999).

19. P. J. McLaughlin, J. T. Gooch, H. G. Mannherz, A. G. Weeds, Structure of gelsolin segment 1-actin complex and the mechanism of filament severing. *Nature* **364**, 685–692 (1993).

20. L. D. Burtnick, D. Urosev, E. Irobi, K. Narayan, R. C. Robinson, Structure of the N-terminal half of gelsolin bound to actin: Roles in severing, apoptosis and FAF. *EMBO J.* **23**, 2713–2722 (2004).

21. R. C. Robinson, M. Mejillano, V. P. Le, L. D. Burtnick, H. L. Yin, S. Choe, Domain movement in gelsolin: A calcium-activated switch. *Science* **286**, 1939–1942 (1999).

22. K. R. Barrie, G. Rebowski, R. Dominguez, Mechanism of actin filament severing and capping by gelsolin. *Nat. Struct. Mol. Biol.* **32**, 237–242 (2025).

23. H. Choe, L. D. Burtnick, M. Mejillano, H. L. Yin, R. C. Robinson, S. Choe, The calcium activation of gelsolin: Insights from the 3A structure of the G4-G6/actin complex. *J. Mol. Biol.* **324**, 691–702 (2002).

24. H. Wang, R. C. Robinson, L. D. Burtnick, The structure of native G-actin. *Cytoskeleton* **67**, 456–465 (2010).

25. P. J. Carman, K. R. Barrie, G. Rebowski, R. Dominguez, Structures of the free and capped ends of the actin filament. *Science* **380**, 1287–1292 (2023).

26. S. Z. Chou, T. D. Pollard, Mechanism of actin polymerization revealed by cryo-EM structures of actin filaments with three different bound nucleotides. *Proc. Natl. Acad. Sci. U.S.A.* **116**, 4265–4274 (2019).

27. N. Li, S. Chen, K. Xu, M.-T. He, M.-Q. Dong, Q. C. Zhang, N. Gao, Structural basis of membrane skeleton organization in red blood cells. *Cell* **186**, 1912–1929.e18 (2023).

28. W. Oosterheert, M. Boiero Sanders, J. Funk, D. Prumbaum, S. Raunser, P. Bieling, Molecular mechanism of actin filament elongation by formins. *Science* **384**, eadn9560 (2024).

29. T. Oda, M. Iwasa, T. Aihara, Y. Maeda, A. Narita, The nature of the globular- to fibrous-actin transition. *Nature* **457**, 441–445 (2009).

30. Y. Kanematsu, A. Narita, T. Oda, R. Koike, M. Ota, Y. Takano, K. Moritsugu, I. Fujiwara, K. Tanaka, H. Komatsu, T. Nagae, N. Watanabe, M. Iwasa, Y. Maéda, S. Takeda, Structures and mechanisms of actin ATP hydrolysis. *Proc. Natl. Acad. Sci. U.S.A.* **119**, e2122641119 (2022).

31. M. Boiero Sanders, W. Oosterheert, O. Hofnagel, P. Bieling, S. Raunser, Phalloidin and DNase I-bound F-actin pointed end structures reveal principles of filament stabilization and disassembly. *Nat. Commun.* **15**, 7969 (2024).

32. L. Jepsen, D. Sept, Effects of nucleotide and end-dependent actin conformations on polymerization. *Biophys. J.* **119**, 1800–1810 (2020).

33. M. G. Saunders, J. Tempkin, J. Weare, A. R. Dinner, B. Roux, G. A. Voth, Nucleotide regulation of the structure and dynamics of G-actin. *Biophys. J.* **106**, 1710–1720 (2014).

34. V. Zsolnay, H. H. Katkar, S. Z. Chou, T. D. Pollard, G. A. Voth, Structural basis for polarized elongation of actin filaments. *Proc. Natl. Acad. Sci. U.S.A.* **117**, 30458–30464 (2020).

35. N. J. Palmer, K. R. Barrie, R. Dominguez, Mechanisms of actin filament severing and elongation by formins. *Nature* **632**, 437–442 (2024).

36. D. J. Kwiatkowski, P. A. Janmey, H. L. Yin, Identification of critical functional and regulatory domains in gelsolin. *J. Cell Biol.* **108**, 1717–1726 (1989).

37. C. Akıl, L. T. Tran, M. Orhant-Prioux, Y. Baskaran, Y. Senju, S. Takeda, P. Chotchuang, D. Muengsaen, A. Schulte, E. Manser, L. Blanchoin, R. C. Robinson, Structural and biochemical evidence for the emergence of a calcium-regulated actin cytoskeleton prior to eukaryogenesis. *Commun. Biol.* **5**, 890 (2022).

38. A. R. Huehn, J. P. Bibeau, A. C. Schramm, W. Cao, E. M. De La Cruz, C. V. Sindelar, Structures of cofilin-induced structural changes reveal local and asymmetric perturbations of actin filaments. *Proc. Natl. Acad. Sci. U.S.A.* **117**, 1478–1484 (2020).

39. K. Tanaka, S. Takeda, K. Mitsuoka, T. Oda, C. Kimura-Sakiyama, Y. Maeda, A. Narita, Structural basis for cofilin binding and actin filament disassembly. *Nat. Commun.* **9**, 1860 (2018).

40. D. M. Mwangangi, E. Manser, R. C. Robinson, The structure of the actin filament uncapping complex mediated by twinfilin. *Sci. Adv.* **7**, eabd5271 (2021).

41. T. J. Mickel, J. J. Childress, Effects of pressure and temperature on the EKG and heart rate of the hydrothermal vent crab Bythograea thermydron (Brachyura). *Biol. Bull.* **162**, 70–82 (1982).

42. D. Simms, P. E. Cizdziel, P. Chomczynski, TRIzol: A new reagent for optimal single-step isolation of RNA. *Focus* **15**, 532–535 (1993).

43. S. Lamble, E. Batty, M. Attar, D. Buck, R. Bowden, G. Lunter, D. Crook, B. El-Fahmawi, P. Piazza, Improved workflows for high throughput library preparation using the transposome-based nextera system. *BMC Biotechnol.* **13**, 104 (2013).

44. M. G. Grabherr, B. J. Haas, M. Yassour, J. Z. Levin, D. A. Thompson, I. Amit, X. Adiconis, L. Fan, R. Raychowdhury, Q. Zeng, Z. Chen, E. Mauceli, N. Hacohen, A. Gnirke, N. Rhind, F. di Palma, B. W. Birren, C. Nusbaum, K. Lindblad-Toh, N. Friedman, A. Regev, Full-length transcriptome assembly from RNA-Seq data without a reference genome. *Nat. Biotechnol.* **29**, 644–652 (2011).

45. S. Nag, Q. Ma, H. Wang, S. Chumnarnsilpa, W. L. Lee, M. Larsson, B. Kannan, M. Hernandez-Valladares, L. D. Burtnick, R. C. Robinson, Ca^{2+} binding by domain 2 plays a critical role in the activation and stabilization of gelsolin. *Proc. Natl. Acad. Sci. U.S.A.* **106**, 13713–13718 (2009).

46. Z. Otwinowski, W. Minor, Processing of X-ray diffraction data collected in oscillation mode. *Methods Enzymol.* **276**, 307–326 (1997).

47. C. Akil, R. C. Robinson, Genomes of Asgard archaea encode profilins that regulate actin. *Nature* **562**, 439–443 (2018).

48. J. W. Brown, J. D. Farelli, C. J. McKnight, On the unyielding hydrophobic core of villin headpiece. *Protein Sci.* **21**, 647–654 (2012).

49. P. Emsley, B. Lohkamp, W. G. Scott, K. Cowtan, Features and development of Coot. *Acta Crystallogr. D Biol. Crystallogr.* **66**, 486–501 (2010).

50. P. D. Adams, P. V. Afonine, G. Bunkoczi, V. B. Chen, N. Echols, J. J. Headd, L. W. Hung, S. Jain, G. J. Kapral, R. W. Grosse Kunstleve, A. J. McCoy, N. W. Moriarty, R. D. Oeffner, R. J. Read, D. C. Richardson, J. S. Richardson, T. C. Terwilliger, P. H. Zwart, The Phenix software for automated determination of macromolecular structures. *Methods* **55**, 94–106 (2011).

51. PyMOL, version 1.2, Schrödinger, LLC.

52. E. Krissinel, K. Henrick, Inference of macromolecular assemblies from crystalline state. *J. Mol. Biol.* **372**, 774–797 (2007).

53. M. J. Abraham, T. Murtola, R. Schulz, S. Páll, J. C. Smith, B. Hess, E. Lindahl, GROMACS: High performance molecular simulations through multi-level parallelism from laptops to supercomputers. *SoftwareX* **1-2**, 19–25 (2015).

54. D. Van Der Spoel, E. Lindahl, B. Hess, G. Groenhof, A. E. Mark, H. J. C. Berendsen, GROMACS: Fast, flexible, and free. *J. Comput. Chem.* **26**, 1701–1718 (2005).

55. J. Lee, X. Cheng, J. M. Swails, M. S. Yeom, P. K. Eastman, J. A. Lemkul, S. Wei, J. Buckner, J. C. Jeong, Y. Qi, S. Jo, V. S. Pande, D. A. Case, Charles L Brooks III, A. D. Mac Kerell Jr, J. B. Klauda, W. Im, CHARMM-GUI input generator for NAMD, GROMACS, AMBER, OpenMM, and CHARMM/OpenMM simulations using the CHARMM36 additive force field. *J. Chem. Theory Comput.* **12**, 405–413 (2016).

56. S. Jo, T. Kim, V. G. Iyer, W. Im, CHARMM-GUI: A web-based graphical user interface for CHARMM. *J. Comput. Chem.* **29**, 1859–1865 (2008).

57. B. R. Brooks, C. L. Brooks III, A. D. Mackerell Jr., L. Nilsson, R. J. Petrella, B. Roux, Y. Won, G. Archontis, C. Bartels, S. Boresch, A. Caflisch, L. Caves, Q. Cui, A. R. Dinner, M. Feig, S.

Fischer, J. Gao, M. Hodoscek, W. Im, K. Kuczera, T. Lazaridis, J. Ma, V. Ovchinnikov, E. Paci, R. W. Pastor, C. B. Post, J. Z. Pu, M. Schaefer, B. Tidor, R. M. Venable, H. L. Woodcock, X. Wu, W. Yang, D. M. York, M. Karplus, CHARMM: The biomolecular simulation program. *J. Comput. Chem.* **30**, 1545–1614 (2009).

58. J. Huang, S. Rauscher, G. Nawrocki, T. Ran, M. Feig, B. L. de Groot, H. Grubmüller, A. D. MacKerell, CHARMM36m: An improved force field for folded and intrinsically disordered proteins. *Nat. Methods* **14**, 71–73 (2017).

59. D. C. Rapaport, *The Art of Molecular Dynamics Simulation* (Cambridge University Press, ed. 2, 2004); www.cambridge.org/core/product/57D40C5ECE9B7EA17C0E77E7754F5874.

60. G. Bussi, D. Donadio, M. Parrinello, Canonical sampling through velocity rescaling. *J. Chem. Phys.* **126**, 014101 (2007).

61. M. Bernetti, G. Bussi, Pressure control using stochastic cell rescaling. *J. Chem. Phys.* **153**, 114107 (2020).

62. B. Hess, H. Bekker, H. J. C. Berendsen, J. G. E. M. Fraaije, LINCS: A linear constraint solver for molecular simulations. *J. Comput. Chem.* **18**, 1463–1472 (1997).

63. L. Verlet, Computer "experiments" on classical fluids. I. Thermodynamical properties of Lennard-Jones molecules. *Phys. Rev.* **159**, 98–103 (1967).

64. T. Darden, D. M. York, N. L. Pedersen, Particle mesh Ewald: An N·log(N) method for Ewald sums in large systems. *J. Chem. Phys.* **98**, 10089–10092 (1993).

65. T. E. I. Cheatham, J. L. Miller, T. Fox, T. A. Darden, P. A. Kollman, Molecular dynamics simulations on solvated biomolecular systems: The particle mesh Ewald method leads to stable trajectories of DNA, RNA, and proteins. *J. Am. Chem. Soc.* **117**, 4193–4194 (1995).

66. M. A. Rould, Q. Wan, P. B. Joel, S. Lowey, K. M. Trybus, Crystal structures of expressed non-polymerizable monomeric actin in the ADP and ATP states. *J. Biol. Chem.* **281**, 31909–31919 (2006).

67. L. R. Otterbein, P. Graceffa, R. Dominguez, The crystal structure of uncomplexed actin in the ADP state. *Science* **293**, 708–711 (2001).

68. L. Holm, L. M. Laakso, Dali server update. *Nucleic Acids Res.* **44**, W351–W355 (2016).
